# RNA Polymerase III Promoters Compatible with CRISPR Gene Regulation in *Saccharomyces cerevisiae*

**DOI:** 10.1021/acssynbio.5c00122

**Published:** 2025-08-18

**Authors:** Kendreze L. Holland, Ines Blancher, Marisa McKesey, Michael Silas, Siddhant Gandhi, Axum Nickerson, Kennedy Jackson, John Blazeck

**Affiliations:** † Department of Biomedical Engineering, 1372 Georgia Institute of Technology, Atlanta, Georgia 30332, United States; ‡ Bioengineering Program, Georgia Institute of Technology, Atlanta, Georgia 30332, United States; § School of Chemical and Biomolecular Engineering, Georgia Institute of Technology, Atlanta, Georgia 30332, United States; ∥ Parker H. Petit Institute of Bioengineering and Bioscience, Georgia Institute of Technology, Atlanta, Georgia 30332, United States; ⊥ Integrated Cancer Research Center, Georgia Institute of Technology, Atlanta, Georgia 30332, United States; # Georgia Immunoengineering Consortium, Emory University and Georgia Institute of Technology, Atlanta, Georgia 30332, United States; ¶ Winship Cancer Institute, Emory University, Atlanta, Georgia 30332, United States

**Keywords:** CRISPR, pol III promoter, yeast, cross-species function, transcriptional regulation, tRNA

## Abstract

*Saccharomyces cerevisiae*is a model organism commonly used to study gene regulation and function recently via CRISPR-(*d*)­Cas9 technologies. Modulating the expression of multiple gene targets simultaneously is often important for synthetic biology and metabolic engineering applications and is crucial for genetic interaction studies. CRISPR-based systems can be used to target multiple genetic loci via expression of multiple single-guide RNAs (sgRNAs) in a single cell. However, there are currently a limited number of well-characterized RNA polymerase III (Pol III) promoters (e.g., pSNR52) for sgRNA expression in *S. cerevisiae*. Herein, we characterize 20 RNA Pol III promoters from different yeast species, from *S. cerevisiae* itself or from mammals, for their utility toward effectively mediating CRISPR activation and repression in *S. cerevisiae*. We show that the Pol III promoter cross-species functionality is impacted by promoter architecture and inclusion of core sequence motifs and that scaffold-mediated recruitment of multiple effectors can rescue poor promoter function in some contexts. Also, we highlight two *Kluyveromyces lactis* Pol III promoters that mediate CRISPR function as well as the gold standard *S. cerevisiae* pSNR52 and previously described tRNA promoters. Finally, we show that these non-native promoters enable effective simultaneous CRISPR-mediated activation and repression of endogenous *S. cerevisiae* genes to enhance resistance to hydrogen peroxide. The Pol III promoters described here highlight the cross-species compatibility of genetic units in simple eukaryotes and will be useful for synthetic biology and phenotype engineering applications in yeast.

## Graphical abstract

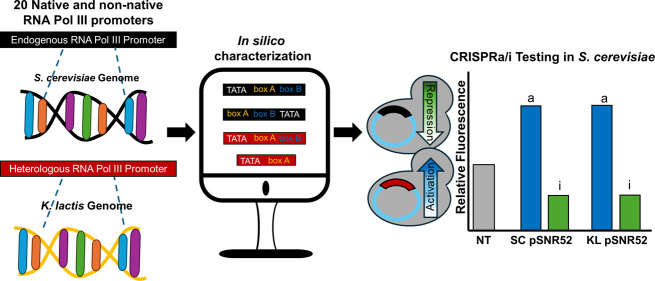

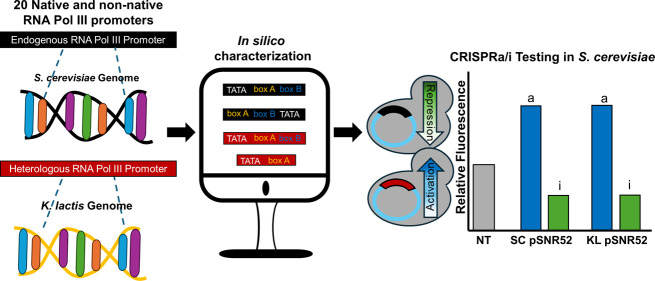

## Introduction

*Saccharomyces cerevisiae*, or yeast, is a simple eukaryotic model organism routinely utilized for genetic studies, as well as industrial biotechnology and metabolic engineering applications.^1–16^ 20–30% of *S. cerevisiae* coding genes have homologues in humans, and several nonconventional yeast species, including *Candida albicans* (30–40%), *Yarrowia lipolytica* (20–30%), and both *Ashbya gossypii* and *Kluyveromyces lactis* (>50%), have similar or higher levels of homology with *S. cerevisiae* with expected conservation of gene function.^17–20^

CRISPR/Cas genetic targeting has been employed for many applications in *S. cerevisiae*, including gene deletion, integration, and editing.^21–23^ Cas9-mediated gene deletions are facilitated by coexpression of a codon-optimized Cas9 endonuclease, often *Streptococcus pyogenes* Cas9, and single-guide RNAs (sgRNAs) with complementarity to a genetic target.^24^ Similarly, deactivated Cas9 (dCas9) can be used to mediate transcriptional activation (CRISPRa) and repression (CRISPRi) by either directly fusing the dCas9 protein to transcription factor domains or by recruiting these domains through aptamers embedded in the sgRNA scaffold.^25,26^ CRISPRa in yeast typically employs a tripartite VP64, p65, Rta (VPR) fusion activating domain, while CRISPRi typically utilizes a MXI1 repressor domain.^25,27^ Scaffolded sgRNAs typically contain MS2 or PP7 stem loops to allow recruitment of multiple effector proteins (that have been fused to stem loop binding proteins) per sgRNA.^28–30^ For instance, Zalatan et al. first used a single dCas9 protein and expression of multiple, distinct scaffold sgRNAs to simultaneously activate or repress expression of VioABEDC genes, generating multicolored phenotypes.^31^

RNA polymerase III (Pol III) promoters, particularly type II Pol III promoters, are often utilized for the expression of sgRNAs in yeast cells. Transcriptional initiation involves recruitment of Pol III complexes, including TFIIIB and TFIIIC, via their association with the Pol III promoter’s “box A” and “box B” motifs.^32,33^ An analysis of yeast tRNA genes, which contain internal Pol III promoters, revealed box A and box B consensus sequences to be TRGYnnAnnnG and GWTCRAnnC, respectively.^34^ Prior studies that have characterized Pol III promoter architecture, including the positioning and specific nucleotide sequences of the TATA box, box A, and box B, have generally focused on the impact on expression of small RNAs, tRNAs, and other nonprotein-coding RNAs.^35–43^ To date, few native Pol III promoters, mainly pSNR52 and less commonly pRPR1,^15,21,44–46^ have been used for sgRNA expression in *S. cerevisiae*, and cross-species utilization of Pol III promoters has not been thoroughly tested in *S. cerevisiae*.^15,21,44,46^ Interestingly, a recent study in yeast characterized tRNA promoters that could increase sgRNA production and processing with the addition of a hepatitis delta virus (HDV) ribozyme to improve Cas9-based genome editing.^47^

Prior studies across eukaryotes have established the benefits of being able to regulate the function or expression of multiple genes in a single cell, for instance, for discovery of complex gene networks, identification of genetic interactions, and metabolic or bioindustrial engineering applications.^48–73^ To enable multigene targeting utilizing CRISPR-(*d*)­Cas9, multiple sgRNAs must be coexpressed in the same cell. In mammalian cells, different species’ U6 RNA Pol III promoters (e.g., human and mouse pU6) have been employed for multi-sgRNA expression to avoid recombination-mediated collapse of the cassette that can occur due to inclusion of repetitive sequence elements.^30,74–76^ As such, access to several “high-functioning” Pol III promoters in yeast could ease CRISPR-(*d*)­Cas9 studies without increasing downstream concerns of multi-sgRNA intracassette recombination.

To this end, we have characterized the ability of 20 Pol III promoters from 7 *s*pecies, in addition to native tRNA promoters and variations that appended a self-cleaving HDV ribozyme, to mediate CRISPR-dCas9 transcriptional perturbations in *S. cerevisiae*. Importantly, we characterized Pol III promoter functionality in the context of both dCas9-mediated transcriptional activation and transcriptional repression of reporter genes across several sgRNAs, as well as utilizing dCas9-activator/repressor fusion proteins or scaffolded-sgRNA recruitment of transcriptional effectors. We observed that the functionality of Pol III promoters varied depending on several factors, including length, the presence of box A and box B sequences, position of the TATA box relative to the box A/B elements, and prevalence of mismatches in the box A and box B sequences compared to consensus sequences. We observed that promoters lacking one or both box A and box B elements, exhibiting significant mismatches in these sequences or with these elements upstream (instead of downstream) of the TATA box, had significantly poorer function in *S. cerevisiae*. We characterized two promoters from*K. lactis*, KL pSNR52 and KL pRPR1, that performed as well or better than the native yeast pSNR52 promoter. In addition, these promoters demonstrated CRISPR functionality equal to or exceeding that of previously described tRNA promoters with or without HDV additions.^47^ As a proof of concept, we employed these strong, cross-species-compatible promoters to simultaneously activate and repress the expression of genes associated with reactive oxygen species stress, resulting in an enhanced cellular phenotype characterized by improved resistance of *S. cerevisiae* to hydrogen peroxide. Finally, with the intention of reducing the cassette size and nucleotide repetition when multiple sgRNAs are expressed in a single construct, we confirmed that these novel promoters functioned toward enabling (d)­Cas9-transcriptional control via separate expression of the crisprRNA (crRNA) and *trans*-activating RNA (tracrRNA) components of an sgRNA. In all, the Pol III promoters characterized in this work can be of general use for yeast engineering, with particular utility for studies requiring transcriptional perturbation.^77^

## Results

### Characterizing Native and Heterologous Pol III Promoters Using a CRISPR Assay

We first engineered *S. cerevisiae* BY4742 (*MAT*α *his3*Δ*1 leu2*Δ*0 lys2*Δ*0 ura3*Δ*0*) strains to constitutively express dCas9 or dCas9-fusion proteins and fluorescent reporters by integrating cassettes into the well-characterized YORWΔ22 and YPRCτ3 genetic loci (Table 1).^78^ A “CRISPRa” test strain (KH5F1) harbored a pTDH3-dCas9-VPR expression cassette as well as the mTagBFP2 fluorescent protein under the control of a weak native constitutive RNA polymerase II promoter (pHTA1) to allow for detection of increased BFP fluorescence via flow cytometry upon sgRNA expression. To the best of our knowledge, only three Pol III promoters from *S. cerevisiae* (i.e., SC pSNR52, SC pRPR1, and SC pZOD1) have been used previously for sgRNA expression.^33,79,80^ Therefore, as a proof of concept, we tested these three promoters alongside four other *S. cerevisiae* Pol III promoters (SC pU6, SC pRNA170, SC pSCR1, and SC pSCR1-extended), as well as the commonly used human and murine pU6 promoters, for the ability to enhance BFP fluorescence in our CRISPRa strain.^15,81–83^ Each Pol III promoter was used to drive the expression of an sgRNA that targeted the pHTA1 promoter (Figure 1A), and we compared the resulting fold increase in fluorescence relative to cells harboring a nontargeting sgRNA. The three previously characterized native promoters, SC pSNR52 (2.3X), SC pRPR1 (2.2X), and SC pZOD1 (2.2X), outperformed the newly tested SC promoters (1.4–1.7X) and the mouse pU6 (1.6X) and human pU6 promoters (1.2X) (Figure 1B).

**Table 1:** Description of Strains Constructed in This Work^a^

strain	base	modifications to base strain
	BY4742	MATα his3Δ1 leu2Δ0 lys2Δ0 ura3Δ0
KH4	BY4742	YORWΔ22Δ:: pTDH3-dCas9-MXI1-tPTP3, psmTEF1-snNAT-tCYC1
KH4F	KH4	YPRCτ 3Δ:: pTDH3-yEGFP-tVMA2, pTEF1-mRuby2-tYHI9, pPGK1-mTagBFP2-tACT1, pFBA1-Hygromycin B-tDDP1
KH4F1	KH4	YPRCτ 3Δ:: pAgTEF1-yEGFP-tVMA2, pHTA1-mTagBFP2-tACT1, pFBA1-Hygromycin B-tDDP1
KH5	BY4742	YORWΔ22Δ:: pTDH3-dCas9-VPR-tPTP1, psmTEF1-snNAT-tCYC1
KH5F1	KH5	YPRCτ 3Δ:: pAgTEF1-yEGFP-tVMA2, pHTA1-mTagBFP2-tACT1, pFBA1-Hygromycin B-tDDP1
KH7	BY4742	YORWΔ22Δ:: pTDH3-dCas9-tRPLS15a, psmTEF1-snNAT-tCYC1, pTEF1-MCP-VPR-tPTP3, pPGK1-PCP-MXI1-tYHI9
KH7F1	KH7	YPRCτ 3Δ::pAgTEF1-yEGFP-tVMA2, pHTA1-mTagBFP2-tACT1, pFBA1-Hygromycin B-tDDP1

a Abbreviations: ag = *Ashbya gossypii*, m = mammalian, d = deactivated, yE = yeast enhanced, sm = *Saccharomyces mikatae*, and sn = *Streptomyces noursei*. All other promoters/terminators are from *Saccharomyces cerevisiae*.

**Figure 1:**
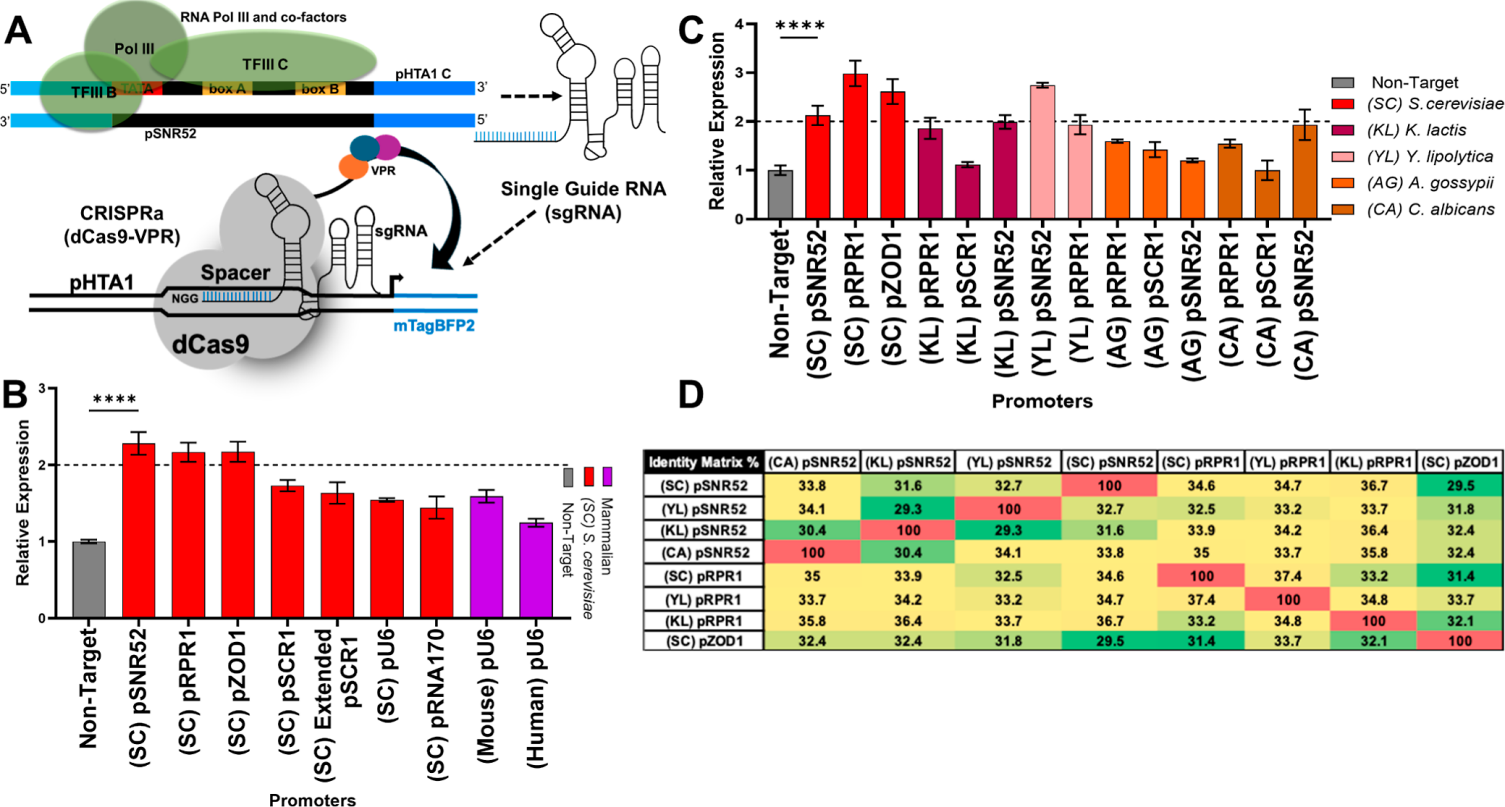
Initial RNA polymerase III (Pol III) promoter characterization. (A) A schematic illustrating the production of sgRNAs and their interaction with deactivated Cas9 (dCas9)-VPR to induce transcription of mTagBFP2. Individual Pol III promoters are used to drive the sgRNA expression in *S. cerevisiae*. (B) Seven *S. cerevisiae* native Pol III promoters and two mammalian U6 Pol III promoters were tested for mediating increased BFP fluorescence in the KH5F1 CRISPRa strain. (C) Eleven additional Pol III promoters from other yeast strains were similarly tested. Promoters that exhibited approximately 2-fold or greater activation were selected for further characterization. (D) Quantification of percent homology between Pol III promoters. Green indicates less homology. In (B,C), error bars represent ±standard deviation of three biological replicates; ****p < 0.0001.

We were interested in whether the presence or absence of box A, box B, and TATA box sequences that match the established *S. cerevisiae* motif sequences might be useful toward predicting the activity of Pol III promoters (Supplemental Figure S1). The *S. cerevisiae* pSNR52, pRPR1, and pZOD1 promoters’ TATA box, box A, and box B motifs have been annotated previously, though pZOD1 lacks a B motif (Supplemental Tables S1–S3).^33,79,80^ We performed an in silico search for each motif in our tested promoters, based on the consensus “TRGYnnARBGG” (box A), “GWTCRAnnC” (box B), and “TATAWAWR” TATA box sequences, in which we noted sequences identical to or up to two mismatches away from the consensus sequence (Supplemental Table S3).^34,84,85^ We observed that the newly tested *S. cerevisiae* Pol III promoters were less defined than pSNR52 and pRPR1, as they lacked box B motifs and contained at least one mismatch from the consensus box A sequence. The human and murine pU6 promoters contained all motifs, though with up to two mismatches from consensus sequences and harboring a different organizational architecture compared to the other tested Pol III promoters. Mainly, the TATA box within the human and murine pU6 promoters, as well as the *S. cerevisiae* pZOD1, was downstream (3′) of the box A/B elements, while most other native yeast promoters harbored an upstream (5′) TATA box sequence. The SC pU6 has a similar organization to human and murine pU6 promoters, suggesting that it is a conserved feature. In addition to SC pSCR1, Pol III promoters that were less than 100 bp in length performed poorly in CRISPRa experiments, suggesting that Pol III promoter length may be important.

Based on this experiment and analyses, we suspected that Pol III promoters (1) have general cross-species functionality, (2) might have higher activity with an “upstream” TATA box organization, and (3) are able to accommodate at least two mutations away from the published consensus sequences of Pol III promoter motifs but could have higher activity with fewer such mutations. Therefore, we next sought to characterize several additional Pol III promoters for their function in *S. cerevisiae*, utilizing sequences from nonconventional yeast species, including *A. gossypii*, *K. lactis*, *Y. lipolytica*, and *C. albicans*. Based on our design rules, we primarily selected candidate promoter sequences that included a TATA box sequence that was “upstream” of box A and box B motifs that contained no more than two mismatches from their consensus sequences. We did include one Pol III promoter, the pSCR1 homologue from *C. albicans*, with the TATA box “downstream” organization as a control. In addition, we made certain that the selected Pol III promoters had limited nucleotide homology to each other and excluded restriction enzymes (i.e., BsaI and *Hin*dIII) used for subcloning. We selected 11 promoters in all for their characterization in comparison to native *S. cerevisiae* promoters.

When tested for their ability to increase fluorescence in our CRISPRa strain, five promoters, KL pRPR1 (1.9×), KL pSNR52 (2.0×), YL pSNR52 (2.7×), YL pRPR1(1.9×), and CA pSNR52 (1.9×), functioned nearly as well as native yeast promoters (Figure 1C). Importantly, all tested promoters had little sequence homology (<38% homology) (Figure 1D and Supplemental Figure S2). Of the weaker promoters, all three selected from *A. gossypii*, the remaining promoter from *K. lactis* (KL pSCR1), and one promoter from *C. albicans* (CA pRPR1) had one to two mismatches from the consensus box A and/or B sequences, while the final weaker promoter, CA pSCR1, harbored the potentially detrimental “downstream TATA box” architecture.

### Characterizing New Yeast-Compatible Pol III Promoters for CRISPRa/i across sgRNA Targeting Sequences

We next characterized our five more promising heterologous promoters for function using multiple sgRNA targeting sequences, as previous studies have highlighted drastic variations in transcriptional perturbations when targeting specific regions of Pol II promoters. We were curious if the general trends we saw for each Pol III promoter’s ability to mediate transcriptional activation would hold across different and potentially less effective sgRNA targeting sequences.^86,87^ Using three sgRNAs that spanned the pHTA1 promoter, 398 (pHTA1-A), 311 (pHTA1-B), and 233 (pHTA1-C—used for initial Pol III promoter characterization) base pairs upstream of mTagBFP2’s start codon (Supplemental Figure S3), we tested each combination of the Pol III promoter and sgRNA in our CRISPRa strain (Figure 2A). We excluded a fourth sgRNA, pHTA1-D, as it targets the promoter on the 3′ side of the TATA box motif, which we confirmed to interfere with transcription (Supplemental Figure S3).^88^ We included the three more active *S. cerevisiae* promoters, pSNR52, pRPR1, and pZOD1, as controls. Surprisingly, only four of the eight total promoters tested, SC pSNR52 and pRPR1 and KL pSNR52 and pRPR1, induced significant BFP fluorescence when driving the expression of more than one of the three sgRNAs, while the remaining four promoters failed to do so for the majority of sgRNAs. Interestingly, SC pZOD1, which has potentially detrimental TATA box organization, performed poorly when using alternative sgRNAs. These results confirm prior work showing that the sgRNA targeting sequence can impact CRISPRa activity and show the importance of the selected Pol III promoter toward overcoming this variability.^89^

**Figure 2:**
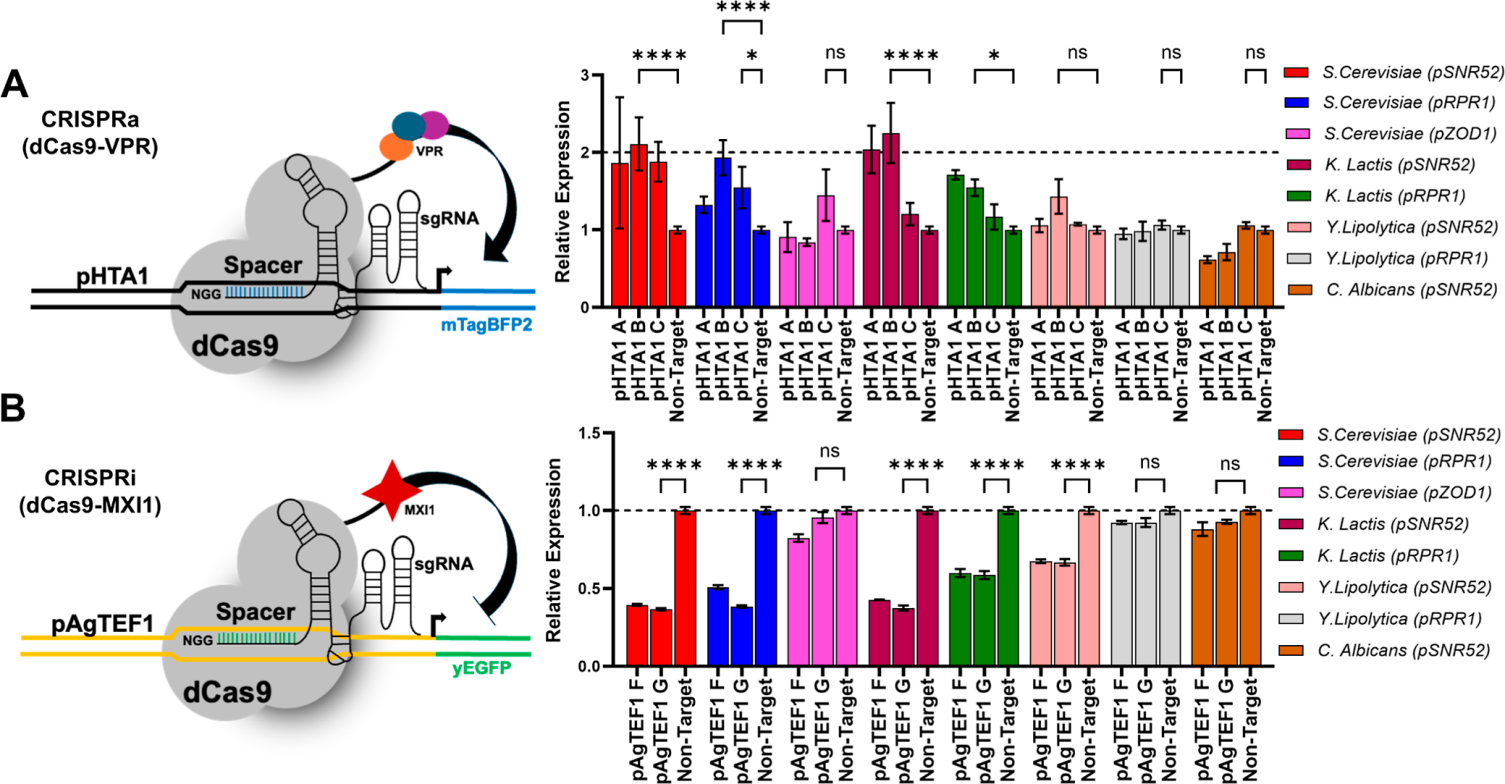
Characterizing native and heterologous Pol III promoters across sgRNAs using CRISPRa/i assays. (A) Eight Pol III promoters were used to drive the expression of three distinct sgRNAs. Resulting fold increases in BFP fluorescence were gauged in the KH5F1 CRISPRa strain. Two non-native Pol III promoters from *K. lactis* enabled significant activation across multiple sgRNAs. (B) The same Pol III promoters were used to drive the expression of two sgRNAs in the KH4F1 CRISPRi strain. Inhibition was gauged via decreased fluorescence of yEGFP, and three non-native promoters showed consistent activity with both sgRNAs. Error bars represent ±standard deviation of three biological replicates; ****p < 0.0001 and *p < 0.05.

We were also interested in the functionality of each Pol III promoter when it was used to express sgRNAs for a different mode of CRISPR-dCas9 transcriptional regulation, mainly CRISPRi. Therefore, we generated a similar “CRISPRi” test strain (KH4F1) harboring pTDH3-dCas9-MXI1 and pAgTEF1-yEGFP expression cassettes to allow facile detection of decreased yEGFP fluorescence upon suitable sgRNA expression.^90^ We characterized our eight promoters of interest in our CRISPRi test strain, again utilizing three targeting sgRNAs spanning the promoter, either 228 (pAgTEF1*-*E), 182 (pAgTEF1*-*F), or 147 (p AgTEF1-G) base pairs upstream of the yEGFP reporter’s start codon (Figure 2B). Interestingly, we saw that five Pol III promoters in all were able to mediate significant transcriptional repression when driving the expression of two sgRNAs, including (once again) SC pSNR52 and pRPR1 and KL pSNR52 and pRPR1 and, newly, YL pSNR52. The sgRNA sequence that targeted the pAgTEF1 promoter farthest from the yEGFP reading frame was nonfunctional in all tests (Supplemental Figure S4).^86,91^

### Comparison of tRNA-Based and Top-Performing Pol III Promoters

We next compared the SC pSNR52, KL pSNR52, and KL pRPR1 promoters to previously described potent tRNA-based Pol III promoters, mainly those of tyrosine (tRNA^Tyr^) and proline (tRNA^Pro^) (Figure 3A).^47^ In addition, each of these five promoters was further modified to include an HDV ribozyme 5′ of the sgRNA sequence, as this addendum has been shown to significantly enhance CRISPR-related functionality for tRNA^Tyr^ and tRNA^Pro 47^. In our CRISPRa test strain, the tRNA promoters, whether modified to include the HDV ribozyme or not, did not significantly reduce the yEGFP fluorescent signal, while SC pSNR52, KL pSNR52, and KL pRPR1 did so as expected (Figure 3B). Addition of the HDV ribozyme reduced these three promoters’ ability to mediate CRISPRi (Figure 3B). In our CRISPRa test strain, the unmodified tRNA^Tyr^ and tRNA^Pro^ promoter did reach significance and were improved by the addition of the HDV ribozyme (Figure 3C). The RNA Pol III promoters, e.g., KL SNR52, had significantly higher BFP signal compared with the tRNA-based constructs, and HDV addition showed a nonsignificant impact (Figure 3C).

**Figure 3:**
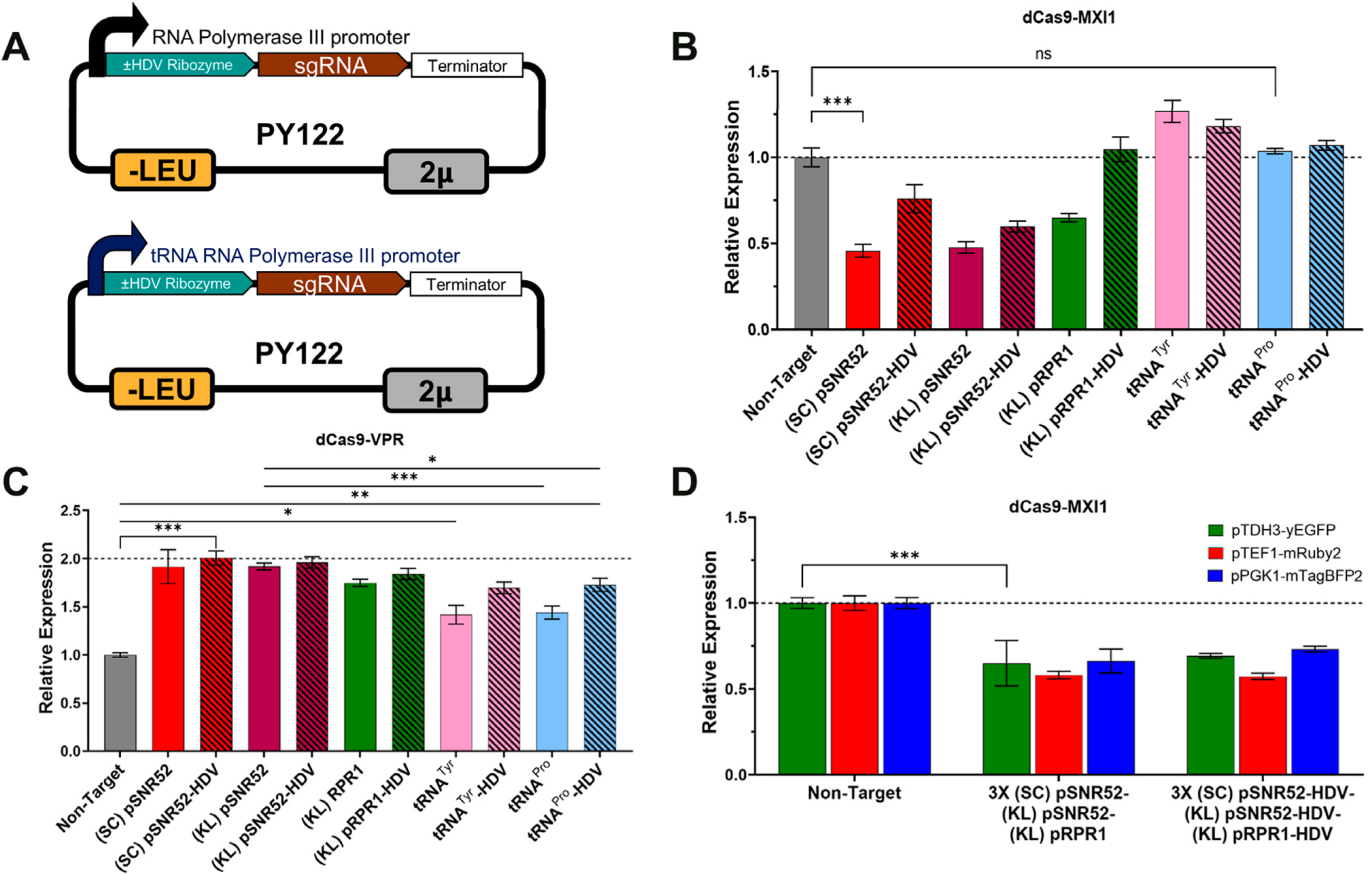
Analysis of tRNA and top-performing Pol III promoters driving the expression of single and multiple sgRNAs in CRISPRa/i assays. (A) The schematic shows two DNA plasmids that express sgRNAs under the control of different promoters (i.e., SC pSNR52 or tRNA^Tyr^), ± HDV ribozyme. (B) In the KH4F1 CRISPRi strain, tRNA promoters failed to reduce the expression of yEGFP via sgRNAs targeting the Pol II AgTEF1 promoter, while native and heterologous Pol III promoters did so. (C) The same promoters were tested in the KH5F1 CRISPRa strain, showing significant activation of BFP by all tested sgRNAs targeting the Pol II pHTA1 promoter. (D) In the three-color KH4F CRISPRi strain, HDV addition did not impact the CRISPRi function of a multi-sgRNA cassette harboring the native *S. cerevisiae* pSNR52 (pos. 1), non-native *K. lactis* pSNR52 (pos. 2), and pRPR1 (pos. 3) promoters. In (B–D), error bars represent ±standard deviation of three biological replicates; ***p < 0.001; **p < 0.01; *p < 0.05; and ns = not significant.

As a prior work with the HDV ribozyme designs often tested them with multi-sgRNA expression, we further assemble multiplex sgRNA cassettes with and without HDV additions.^47^ We first engineered a new 3-clor CRISPRi strain (KH4F), in which the strong pTDH3, pTEF1, and pPGK promoters drove the expression of yEGFP, mRuby2, and mTagBFP2, respectively, and validated sgRNAs for each fluorescent reporter construct (Supplementary Figure S5). Then, we made constructs in which one native and two non-native Pol III promoters, either with or without HDV ribozymes, were used to drive sgRNAs in multi-sgRNA cassettes (Figure 3D). The addition of the HDV ribozyme did not noticeably impact the expression of the three fluorescent reporters.

**Figure 4:**
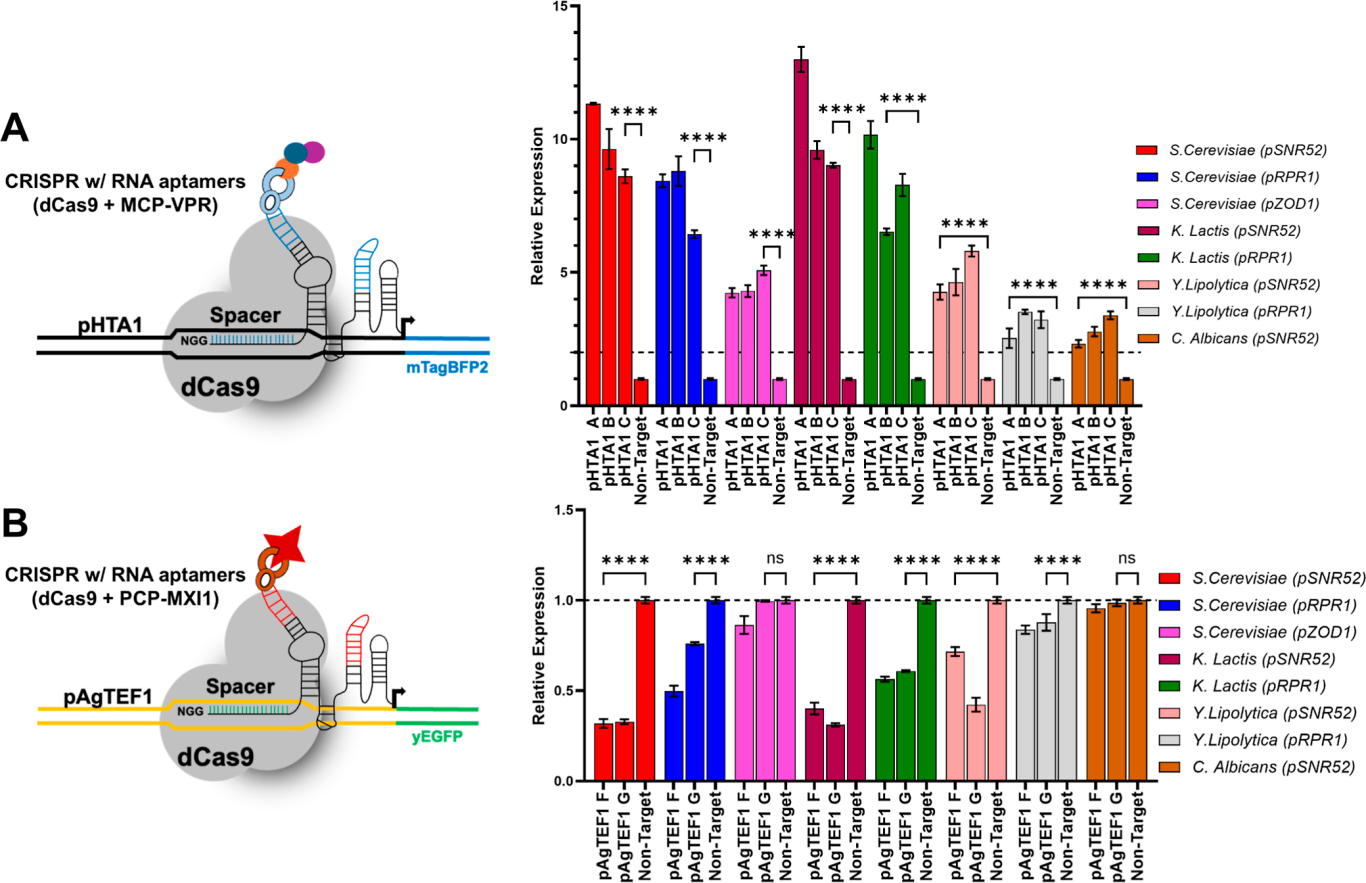
Rescuing RNA Pol III promoter function with scaffolded transcriptional effector recruitment (CRISPR w/RNA aptamers). (A) Eight Pol III promoters driving sgRNA (MS2-scaffolded) production were tested in the yeast strain KH7F1. All promoters showed enhanced gene activation with sgRNAs expressing MS2 RNA aptamers. (B) The same eight Pol III promoters were used to drive PP7-scaffolded expression in the KH7F1 strain. In (A,B), error bars represent ±standard deviation of three biological replicates; ****p < 0.0001.

### Rescuing RNA Pol III promoter function was achieved using CRISPR activation and inhibition mediated by scaffolded transcriptional effector recruitment.

We were surprised that the YL pRPR1 and CA pSNR52 promoters failed to significantly activate or inhibit fluorescent reporter expression when driving the expression of most sgRNAs tested in our initial CRISPRa and CRISPRi strains (Figure 2), as in their initial characterization (Figure 1C), they significantly increased mTagBFP2 expression. Therefore, we were interested in employing different modes of dCas9-based transcriptional regulation to see if we could enhance the transcriptional modulation enabled by these promoters across a range of sgRNAs. Compared to the genetically fused dCas9-VPR or dCas9-MXI1, recruiting RNA-binding proteins complexed to transcriptional activators (e.g., MCP-VPR) or repressors (e.g., PCP-MXI1) via scaffolded sgRNAs embedded with molecular aptamers (MS2 and PP7) has been shown to increase activation or inhibition levels by allowing multiple effectors to complex with a single dCas9–sgRNA complex.^29–31^ We next tested if the function of our weaker Pol III promoters could be rescued in *S. cerevisiae* by employing scaffolded sgRNA CRISPR activation and inhibition platforms. We engineered an *S. cerevisiae* strain (KH7F1) to harbor dCas9, pHTA1-mTagBFP2, and pAgTEF1-yEGFP expression cassettes, as well as integrating expression cassettes for the RNA aptamer binding-activator/inhibitor fusion proteins, MCP-VPR and PCP-MXI1.^16,92^ We added relevant MS2 aptamer sequences into the first and third loops of the pHTA1-A, B,C sgRNA sequences, and PP7 aptamers into the same loops of the pAgTEF1-E, F,G sgRNA sequences, and then tested eight Pol III promoters for their ability to activate or repress fluorescence in this scaffolded CRISPR activation and inhibition system (Figure 4).^31^ All eight promoters afforded significant activation of the mTagBFP2 fluorescence (Figure 4A), reaching up to 13-fold for the KL pSNR52 promoter driving the expression of the pHTA1-A sgRNA. Even the SC pZOD1, YL pSNR52, YL pRPR1, and CA pSNR52 promoters that performed poorly in the standard CRISPRa strain were able to afford statistically significant increases in BFP expression when used to drive the expression of any of the three tested pHTA1 targeting sgRNAs, indicating that the ability to recruit multiple transcriptional activator proteins can generally rescue the functionality of the Pol III promoters for CRISPR activation. However, the use of PP7-scaffolded sgRNAs (and coexpression of PCP-MXI1) did not benefit SC pZOD1, YL pRPR1, or CA pSNR52 for CRISPR inhibition (Figure 4B).

### Simultaneous CRISPR Activation and Inhibition with Native or Heterologous Pol III Promoters and Coexpression of Separated sgRNA Chimera Components

Having shown the strong functionality of pSNR52 and pRPR1 from *S. cerevisiae* and *K. lactis* across sgRNAs and CRISPR platforms, we wanted to confirm that they could enable multiplexed sgRNA expression to modulate the expression of multiple genes per cell. As a proof of concept, we constructed plasmids with dual sgRNA cassettes in which pSNR52 (from *S. cerevisiae* or *K. lactis*) drove the expression of the pHTA1-C sgRNA harboring MS2 aptamers and pRPR1 (again from either *S. cerevisiae* or *K. lactis*) drove the expression of the pAgTEF1-F sgRNA harboring PP7 aptamers. These plasmids, which we expected to be able to enable simultaneous activation and inhibition of the two fluorescent reporters, were then transformed into the yeast strain KH7F1. As hoped, the heterologous KL pSNR52 and pRPR1 mediated CRISPR activation and inhibition, as well as the native *S. cerevisiae* Pol III promoters (Figure 5). We further confirmed that switching the positions of the aptamers in the sgRNAs or the sgRNA and promoter order within the dual sgRNA cassettes had no impact on CRISPR activation and inhibition of the fluorescent reporters (Supplemental Figure S6). We also demonstrated that KL pSNR52 and KL pRPR1 demonstrated comparable or superior transcriptional activation and/or repression, in both single sgRNA and two-sgRNA multiplex cassette configurations, than observed with tRNA^Tyr^ and tRNA^Pro^ ± HDV ribozyme designs (Supplemental Figure S7).

**Figure 5:**
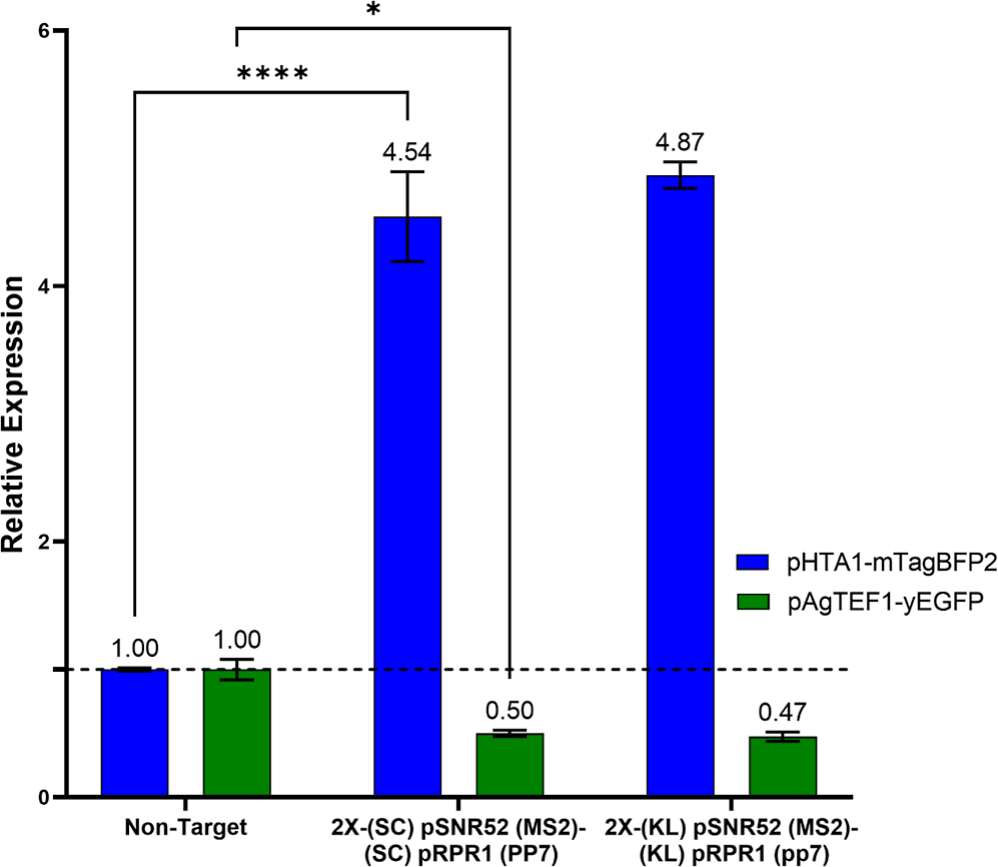
Simultaneous activation and inhibition of fluorescent reporter genes. Comparison of simultaneous transcriptional activation and inhibition by Pol III promoters from *S. cerevisiae* and *K. lactis* *.* The values above each bar represent the relative expression of the fluorescent reporter, with raw data normalized to the nontargeting control. Values greater than 1 indicate activation of mTagBFP2, while values less than 1 indicate repression of yEGFP. These experiments were performed in yeast strain KH7F1, and error bars represent ±standard deviation of three biological replicates; ****p < 0.0001; and *p < 0.05.

While using different Pol III promoters to drive the expression of each sgRNA in a multi-sgRNA cassette reduces the potential repetition of nucleotide sequences, the sgRNA itself will still contain repeated sequences. Larger multi-sgRNA cassettes, particularly those harboring aptamer sequences, may still contain enough repeated DNA stretches to cause instability in yeast.^93^ Therefore, we next demonstrated that separate coexpression of the components of the chimeric sgRNA (the targeting crRNA and the tracrRNA) using our high-functioning native or heterologous Pol III promoters enabled transcriptional activation in both of our CRISPRa platform strains, i.e., via dCas9-VPR fusion or via scaffolded tracrRNAs and MCP-VPR coexpression (Supplemental Figure S8).^94^ For the latter configuration, these results may also represent the first functional demonstration of separate crRNA and scaffolded tracrRNA expression in yeast.

### Application of High-Functioning Heterologous Pol III Promoters to Increase ROS Resistance

Finally, we sought to engineer a beneficial phenotypic change using simultaneous gene targeting with our newly characterized Pol III promoters in yeast. Reactive oxygen species (ROS) are produced by mitochondria as a byproduct of oxygen transport through the electron transport chain and are also encountered as extracellular stressors in bioindustrial processes. As such, increased resistance to ROS is beneficial for some yeast-based processes. ROS levels are regulated by a network of genes to maintain cellular homeostasis.^54,95^ This regulation primarily involves reducing reactive oxygen molecules to less reactive forms by enzymes like CTT1, cytosolic catalase T.^96^ In addition, single-gene knockout screens have shown that deletion of transketolase (Δ*TKL1*) increases yeast resistance to acute exposure to hydrogen peroxide, a reactive oxygen species.^97^ We created CRISPR activation and inhibition strains in which expression of *CTT1* and *TKL1* was simultaneously activated or repressed, respectively, via coexpression of scaffolded sgRNAs using either high functioning heterologous (KL pSNR52 and KL pRPR1) or native (SC pSNR52 and SC pRPR1) Pol III promoters. After treatment for 1 h with hydrogen peroxide as an exogenous ROS stressor, yeast cells were serially diluted and spotted on plates to assay survival (Figure 6A).^98^ Remarkably, our engineered strains with dual transcriptional perturbations were resistant to all levels of hydrogen peroxide tested (up to 140 mM), while the nonengineered control strain was killed by 50 mM H_2_O_2_. We further validated via RT-qPCR the simultaneous activation and inhibition of the targeted genes, *CTT1* and *TKL1*, with significant mRNA changes for both genes when employing our newly described KL promoters (Figure 6B). The SC promoter-based construct did not reach significance for *TKL1* transcriptional inhibition.

**Figure 6:**
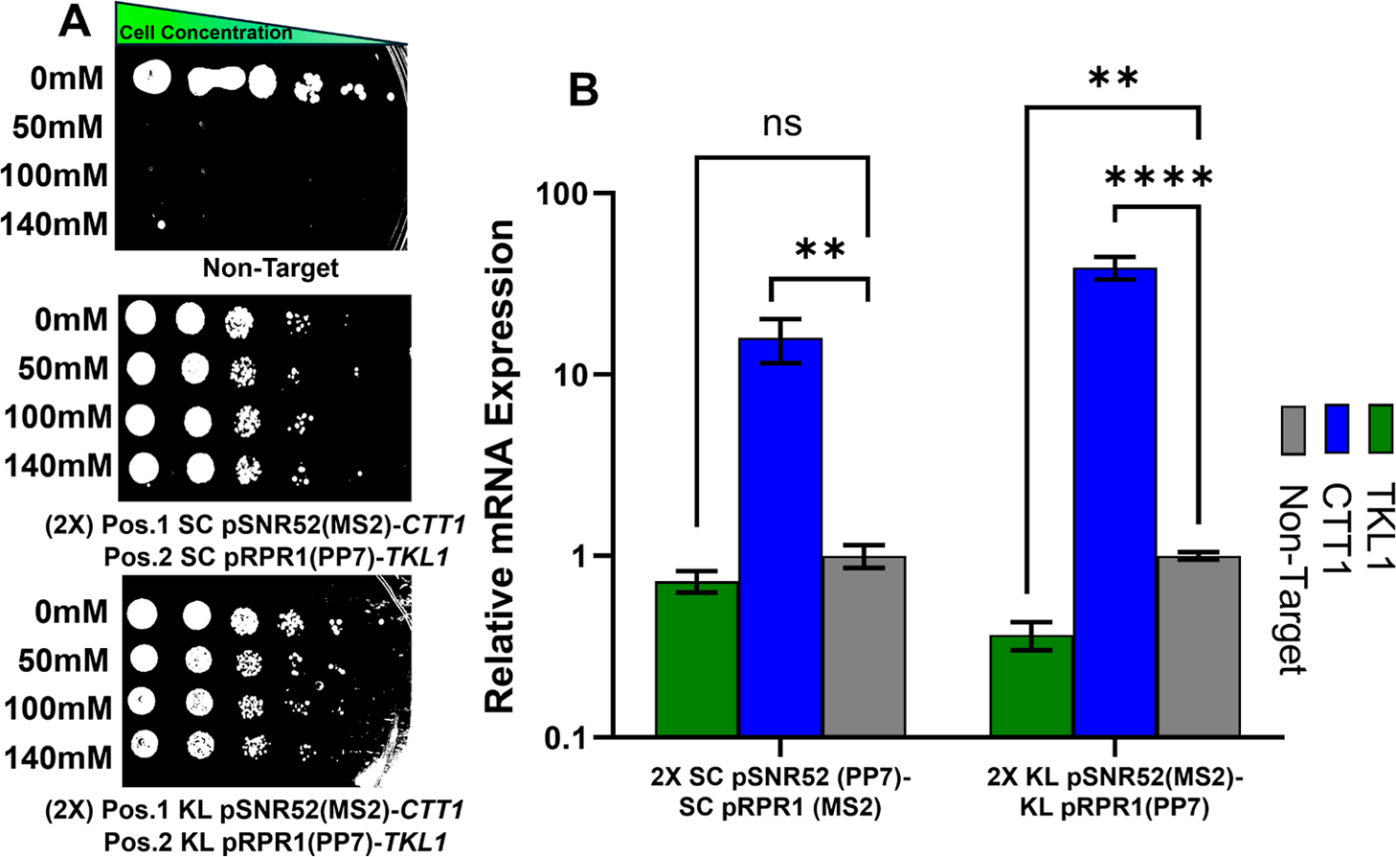
Semiquantitative spot test and RT-qPCR analyses of genes targeted simultaneously to confer resistance to hydrogen peroxide. (A) A spot test validated that KH7F1 cells, transformed with 2× cassettes targeting antioxidant (*CTT1*) and ROS producing (*TKL1*), demonstrated resistance to cytotoxic concentrations (50 mM, 100 mM, and 140 mM) of hydrogen peroxide. (B) Three colonies treated with 140 mM hydrogen peroxide were picked from plates display surviving colonies from the spot test for further quantification of changes in relative mRNA expression by RT-qPCR. These changes resulted from the simultaneous activation and repression of genes targeted by sgRNAs produced by 2× cassettes expressing native (SC pSNR52 and pRPR1) and non-native (KL pSNR52 and pRPR1) Pol III promoters. In (A,B), these experiments were performed in yeast strain KH7F1, error bars represent ±standard deviation of three biological replicates; ****p < 0.0001 and **p < 0.01.

## Discussion

RNA Pol III promoters and tRNAs both contain motifs that facilitate the recruitment of essential transcriptional machinery.^33^ In our studies, we found that the functionality of Pol III promoters in yeast was influenced by several factors, including length, the presence of both box A and box B sequences, the position of the TATA box relative to these two sequences, and the occurrence of mismatches between box A and box B sequences and the consensus sequence. The presence of such elements in tRNA sequences makes them candidates for use for CRISPR applications, though the strongest promoters we characterized outperformed previously described tRNA constructs, either with or without the addition of self-cleaving HDV ribozymes.^99,100^

A potential application of our work is to be able to reduce repetitive sequences in multi-sgRNA cassettes by avoiding reuse of the same Pol III promoter. We note that a promising alternative to our efforts has been reported by Liu et al., i.e., using a sgRNA-tRNA array for multi-sgRNA expression, in this case for multigene disruptions with CRISPR-Cas9.^101^ Their elegant method takes advantage of the intrinsic Pol III promoter activity of intervening tRNAs, as well as native tRNA intracellular processing that releases “mature” sgRNAs.^101–103^ Our efforts to characterize strong Pol III promoters are complementary to these efforts, and we posit that our top performing promoters are ideally suited for future synthetic biology and CRISPR-associated multigene perturbation platforms. Moreover, we demonstrated that we could coexpress separate components of sgRNAs using different Pol III promoters, further reducing potential nucleotide repetition. In addition, the separate expression and functional validation of a scaffolded tracrRNA and crRNA do not appear to have been reported previously.

It is important to note that the CRISPR-based transcriptional control that we describe can be unstable due to several factors. For instance, cells may lose plasmids over time, and sgRNAs may have off-target effects, resulting in a shift in transcriptional profiles.^104,105^ Additionally, changes in the chromatin state of cells as they progress through the cell cycle can also alter gene expression.^106,107^ As such, it will be of interest to validate the long-term function of heterologous Pol III promoters toward CRISPRa and CRISPRi in the future.

Overall, our work aimed to expand the toolbox for CRISPR-mediated transcriptional perturbations in yeast, which we were able to do by taking advantage of the Pol III promoter cross-species functionality. In general, this cross-species functionality may arise from the tolerance of Pol III promoter elements toward inclusion of nonconsensus nucleotides. The relaxed nature of required sequences even allowed the function of mammalian promoters in our yeast-based activation tests. To further investigate this phenomenon, it would be worthwhile to expand our efforts toward testing for cross-kingdom (e.g., mammalian, plant) Pol III compatibility in yeast, though such exploratory studies may yield poorly functional promoters as divergence in sequences can prevent even interspecies compatibility. In addition, it could be possible to develop synthetic RNA Pol III promoters for yeast. In this vein, an interesting study developed hybrid, synthetic RNA Pol III promoters that functioned well in *Y. lipolytica*. This hybrid promoter, which consists of a truncated native *Y. lipolytica* Pol III promoter fused to a tRNA gene, has been documented to have promoter-like activity in both yeast and mammalian cells.^108,109^

In our work, we also showed that it was possible to “rescue” the function of lower activity Pol III promoters using a scaffolded CRISPRa approach, though a similar scaffolded CRISPRi approach was unsuccessful. This contrast may result from differential mechanisms of action for each effector protein and the resulting ability to mediate increased transcriptional perturbation via increased localization of effectors. VPR, composed of VP64 and two other potent transcriptional activator domains, enhances chromatin accessibility by recruiting additional activators such as histone acetyltransferases (HATS), relaxing chromatin structure.^110^ Previously, multirecruitment of VP64 effectors to synthetic yeast promoters has been shown to enhance transcriptional activation, suggesting an additive effect. In addition, the SunTAG CRISPRa system has been shown to have high activity in mammalian cells by localizing 10 copies of VP64 to a given genetic loci.^111^ In contrast, MXI1, a component of the mSin3/histone deacetylases (HDAC) complex that recruits HDACs to compact chromatin structure to ultimately reducing RNA Polymerase II binding, may lack the same additive effect seen with VP64-based effectors.^112^ In an alternative strategy, it may be possible to “rescue” low-activity Pol III promoters by introducing consensus box A/B sequences, by adjusting their architecture to take the seemingly preferred format, or by taking other guided evolutionary approaches.^113^ *S. cerevisiae* RNA polymerase II promoters have been shown to be amenable toward such strategies.^11,114,115^

## Conclusion

In all, we characterized 20 Pol III promoters from 7 species and then compared top performing promoters to tRNAs and their derivatives that have been shown to be particularly potent. We further analyzed these Pol III promoters’ ability to facilitate gene perturbation using fused or aptamer-recruited effector set ups and across several sgRNA sequences, showing that two heterologous *K. lactis* Pol III promoters had function on par with the most robust native *S. cerevisiae* Pol III promoters tested. These *K. lactis* Pol III promoters were amenable to multiplex sgRNA expression and simultaneous CRISPR activation and inhibition. Finally, we subjected our most effective Pol III promoters to a (literal) stress test by simultaneously activating and repressing genes important for ROS resistance, attaining engineered yeast cells that are able to survive acute exposure to high concentrations of hydrogen peroxide. Our newly characterized Pol III promoters can help expand the biological toolbox in yeast, having applications in genetic studies, industrial biotechnology, and metabolic engineering.

## Methods

### Media, Culture, and Base Strains

Plasmids constructed for experiments were propagated in NEB 10-beta *E. coli* (New England Biolabs), grown in 5 mL of LB broth (Teknova) supplemented with 34 μg/mL chloramphenicol (Sigma-Aldrich) antibiotic for selection. These cells were then cultured overnight at 37 °C and 250 rpm to induce constant agitation. After 16 h of growth, plasmids were extracted using a Qiaprep Spin Miniprep Kit (Qiagen). Plasmids were confirmed by Sanger sequencing.

We used BY4742 (*MAT*α *his3*Δ*1 leu2*Δ*0 lys2*Δ*0 ura3*Δ*0*; ATCC 4040004, YVC1) base strain for integrating the CRISPR-associated protein and fluorescent reporters. Cells transformed with a plasmid were cultured in a started culture of 5 mL of synthetic glucose (dextrose)-Leu media, which consisted of 0.69 g/L complete supplemental media-LEU (CSM-LEU, Sunrise Science), 0.67 g/L yeast nitrogen base (YNB, BD), and 20 g/L of glucose (Fisher Scientific). Then, cells were taken from this media and added to a large volume of synthetic media supplemented with the same reagents. When integrating genes into different chromosomes, YPD media were supplemented with 100 μg/mL nourseothricin (NAT) (Gold Biotechnology) or hygromycin B (Thermo Fisher Scientific) for NAT and Hygromycin B selection, respectively. Cells with integrated genes were then grown in YPD media that only contained 10 g/L yeast extract (Thermo Fisher), 20 g/L peptone (Thermo Fisher), and 20 g/L glucose.

### General Cloning Procedures

DNA fragments were amplified via a polymerase chain reaction with KOD Hot Start DNA polymerase (Sigma-Aldrich) as the initiator. DNA oligomers designed in silico were synthesized and processed by Eurofins Genomics. Primers and oligomers used in this study can be found in Supplemental Tables S4–S6. We prepared our own Gibson mix for Gibson assembly cloning by combining Taq Ligase (Enzymatics), Phusion polymerase (New England Biolabs), and T5 Exonuclease (New England Biolabs). To prepare the Gibson reaction, 100 ng of linearized backbone DNA was combined with a 2× molar excess of PCR inserts in a 5 μL volume. Subsequently, 15 μL of Gibson mix was added, and the reaction was initiated on a thermocycler at 50 °C for an hour. For Golden Gate Assembly, we used a modified protocol to meet our goal of obtaining a large number of transformants post-transformation into NEB 10-beta *E. coli*.^101^ Single oligos with complementary sequences (20 bp spacer with sticky ends) were combined into a PCR tube containing 5 μL of each oligo. Next, 10 μL of water was added to a total volume of 20 μL per reaction. The thermocycle (Bio-Rad) conditions were 97 °C for 5 min and then ramped down to 20 °C over the course of 35 min. Then, 1 μL of anneal oligos was added to 100 ng of base plasmid, 2 μL of T4 Ligase 10× Buffer, 1.5 μL of T4 Ligase (New England Biolabs), and 1 μL of BsaI-HFv2 (New England Biolabs). The following temperature profile was followed for the reaction: step 1, 37 °C for 30 min; step 2, 37 °C for 10 min; step 3, 16 °C for 5 min; step 4, repeat steps 2 and 3 for 30 cycles; step 5, 37 °C for 30 min; step 6, 60 °C for 5 min; step 7, 80 °C for 5 min; and step 8, 4 °C hold. Upon completion of the Golden gate reaction, 12 μL of volume was dialyzed against ultrapure water to remove any salt or reagents that could cause an arc in the subsequent electroporation step. After using the standard electroporation method to insert the DNA plasmids into *E. coli*, they were recovered for 1 h at 37 °C and then plated on solid media with chloramphenicol selection. These plates were grown overnight at 37 or 30 °C. LB cultures were inoculated with colonies isolated from the plate, followed by plasmid extraction and Sanger sequencing.

### gRNA Plasmid Cloning

Non-native Pol III promoter, tRNA, and HDV ribozyme sequences were synthesized by TWIST Bioscience, while *S. cerevisiae* promoters were amplified using PCR primers listed in Supplemental Tables S3–S6 extracted from genomic DNA using a YeaStar Genomic DNA kit (Zymo Research). Single targeted sgRNA plasmids were assembled by using two cloning methods. The first method involved Gibson assembly, in which a PY122 plasmid cut with the BsaI restriction enzyme was combined with PCR-amplified fragments that included the “promoter-20bp spacer” and “20 bp spacer-sgRNA-terminator” (3 fragments) to test in our engineered yeast strain. The second method used both methods, initially to create a Golden-Gate-compatible base plasmid with Gibson to then insert annealed 20 bp oligomers with flanking BsaI cut sites. All initial gRNA scaffolds such as unmodified sgRNA, MS2/PP7 1,3, and 2× tails (4 total) were synthesized by TWIST Biosciences. Two plasmids, PY122 (2 μ, LEU-selection) and PY128 (2 μ, g418 resistance), were used as templates to construct plasmids that expressed a “constitutive Pol III promoter-dummy region-crRNA-terminator” and “constitutive Pol III promoter-tracrRNA-terminator”, respectively, via Gibson assembly. Specifically, each plasmid comprised a constitutive yeast Pol III pSNR52 promoter, a blank gap region bordered by BsaI cut sites (base plasmid), the sgRNA scaffold variant, and a terminator sequence derived from tSUP4 (Supplemental Table S7). The blanked gap regions were replaced by annealed 24 bp oligos assembled by Golden Gate with compatible BsaI cut sites to the digested base plasmid, creating sgRNAs capable of targeting pol II promoters driving protein-encoded genes (i.e., yEGFP). A full list of spacer sequences can be found in Supplemental Table S8. To construct the 2× multi-sgRNA expression cassettes, a linearized PY122 plasmid digested with the BsaI restriction enzyme was combined with two fragments, each amplified with PCR from separate templates. These fragments included the region of interest: “Pol III promoter–20 bp spacer–sgRNA (aptamers)–tSUP4.” The components were assembled using Gibson assembly, resulting in a 2× cassette that expressed sgRNAs, with each regulated by a distinct Pol III promoter on a single DNA construct.

### Yeast Strain Engineering

The engineering of yeast strain KH4F1, which was used during CRISPRi experiments, involved cotransforming linear DNA fragments with >50 bp of homology to the neighboring fragment (e.g., HR1 has homology to HR2 and HR2 has homology to HR3, etc.).^90^ Into a integration loci that affords robust gene expression, linear DNA fragments were integrated into these sites by the high-efficiency, lithium acetate transformation method.^78,116,117^ First, into YORWΔ22, dCas9-MXI1 along with the NAT gene created the strain KH4. Into this new strain, we used a different integration site, YPRCτ 3, to cotransform 2 fluorescent reporters, yEGFP and mTagBFP2, and hygromycin B for selection (KH4F1). In the same base strain, we integrated two fluorescent reporters driven by different constitutive Pol II promoters (pTDH3 and pPGK1), along with a third reporter, mRuby2, regulated by pTEF1, to create the three-color KH4F strain. These integrations were confirmed by PCR of extracted genomic DNA. For creation of strains KH5F1 and KH7F1, the same two integration sites were used to clone dCas9-VPR (CRISPRa) and dCas9/MCP-VPR/PCP-MXI1 (CRISPR activation and inhibition with RNA aptamers; 4 fragment insertion), respectively, into YORWΔ22. The genes integrated into YPRCτ 3 remained the same. Finally, sgRNA plasmids were transformed into desired strains using the same lithium acetate transformation protocol as used for integrations.

### Cell Prep and Flow Cell Analysis

Engineered yeast cells were inoculated into 5 mL cultures of synthetic media (CSM-LEU) 1X plasmids) or CSM-LEU and g418 (2× cassettes). These cells were grown overnight at 30 °C to an OD_600_ of 0.4. These cells were then transferred to smaller FACS tube, spun down, washed with 1× phosphate buffer saline (PBS), and resuspended into 1 mL of 1× PBS. On the FACS Fortessa (BD Sciences), 50,000 events were recorded for detection of changes in yEGFP, mRuby2, and mTagBFP2 expression. Flow cytometry data analysis was performed by using FlowJo.

### Spot Tests

To assess the survival of engineered yeast cells exposed to hydrogen peroxide, we used Tran et al.’*s* protocol for semiquantitative plate spot analysis.^98^ Yeast cells were grown overnight to an optical density (OD_600_) of 0.5 in a starter culture of synthetic media (CSM-LEU) and then transferred to a larger culture, where they were allowed to grow for an additional 6–8 h. Next, these cells were diluted to an optical density (OD_600_) of 5–6 (e.g., 0.3 × 4 (dilution factor) ×4 (instrument error = 4.8) in a 10 mL of synthetic medium (125 mL Erlenmeyer flask). The flasks were then incubated at 30 °C for 1 h in the presence of different concentrations of hydrogen peroxide (Fisher Scientific). After the incubation period, 1 mL of the cells was spun down and washed with 1× phosphate-buffered saline (PBS). The washed cells were then resuspended in 1 mL of fresh 1× PBS. We then performed a 10-fold serial dilution of 200 μL of cells in a 96-well plate, transferring 5 μL from each well onto a CSM-LEU plate.

### RT-qPCR Analysis

Single colonies treated with the highest concentration of hydrogen peroxide (140 mM) from the spot test analysis were selected and grown for 24 h to achieve a high optical density (OD). Following the protocol from the RNeasy Mini Kit (Qiagen), over 20 μg of total RNA was extracted and immediately used for real-time polymerase chain reaction (RT-qPCR) analysis. Using the Luna Universal One-Step RT-qPCR Kit (NEB), total RNA was converted into cDNA, with primers targeting *CTT1*, *TKL1*, and *ACT1* (housekeeping gene). Dilutions of known concentrations (100 to 0.1 ng) of pooled template RNA from tested samples were prepared on a 96-well plate for quantification of cDNA. These dilutions were used to construct a standard curve for quantifying the tested samples to measure changes in mRNA expression induced by sgRNAs capable of activating or inhibiting targeted genes. The cycle conditions for running the 96-well plate on an Applied Biosystems StepOnePlus real-time PCR System (Applied Biosystems) are as follows: (1) reverse transcription at 55 °C for 10 min (1 cycle), (2) initial denaturation at 95 °C for 1 min (1 cycle), and (3) denaturation at 95 °C for 10 s and extension at 60 °C for 1 min (40 cycles). Since the reagents contained SYBR Green, a melt curve was performed, starting at 60 °C and gradually increasing the temperature by 0.3 °C increments to 95 °C for 10 s (1 cycle).

## Statistical Analysis

All the flow cell experimental samples were performed in biological triplicate (*n* = 3). All reported error bars represent one standard deviation. To calculate p-values, a multiple comparison test using Tukey’s range method was done using GraphPad Prism.

## Supplementary Material



## Notes

Promoter and primer sequences; overall promoter analyses; primers for cassettes; gRNA scaffold details; gRNA spacer details; sequences and lengths of RNA polymerase III promoters, characterization of ill-defined Pol III DNA elements, assembly method and primer sequences, and 20 bps targeting sequences; Supplemental Figure S1: Transcription initiation complex interactions with Pol III promoters encoded with optimal and non-ideal, ill-defined DNA elements. Supplemental Figure S2: Attempted alignment of the eight characterized RNA Pol III promoters. Supplemental Figure S3: sgRNAs engineered to target different regions of pol II promoter pHTA1 and pAgTEF1 in yeast strains KH5F1 and KH4F1, respectively. Supplemental Figure S4: Poor repression mediated by an sgRNAs in dCas9-MXI1 and dCas9 + PCP-MXI1 transcriptional regulation systems. Supplemental Figure S5: Comparing 1X sgRNAs driven by native and non-native promoters in a CRISPRi assay. Supplemental Figure S6: Simultaneous activation and repression of fluorescent reporter genes. Supplemental Figure S7: Simultaneous activation and repression of fluorescent reporter genes by promoters with various architectures. Supplemental Figure S8: Expressing crRNA and tracrRNA of a native and modified sgRNA scaffolds on separate yeast expression plasmids. Supplemental Table S1: Sequences and Lengths of RNA Polymerase III promoters in this study. Supplemental Table S2: Efficacy of Native and Cross-Species Promoters in different CRISPR assays. Supplemental Table S3: Characterization of ill-defined Pol III DNA elements. Supplemental Table S4: Assembly Method and Primer Sequences for 1X Pol III Promoter cassettes with standard sgRNAs. Supplemental Table S5: Assembly Method and Primer Sequences for 1X Pol III Promoter cassettes and Aptamer-scaffolded sgRNAs. Supplemental Table S6: Assembly Method and Primer Sequences for 2X and 3X Pol III Promoter cassettes and Aptamer-scaffolded sgRNAs. Supplemental Table S7: Assembly Method and Primer Sequences for 1X Pol III Promoter cassettes with Separate crRNA and tracrRNAs. Supplemental Table S8: 20 bps Targeting Sequences with BsaI Overhangs and RT-qPCR primers (PDF)

## References

[1] Liu W. (2017). From Saccharomyces cerevisiae to human: The important gene co-expression
modules. Biomed Rep.

[2] McGary K. L., Lee I., Marcotte E. M. (2007). Broad network-based predictability of Saccharomyces
cerevisiae gene loss-of-function phenotypes. Genome Biol..

[3] Montpetit B. (2005). Genome-Wide Synthetic
Lethal Screens Identify an Interaction Between
the Nuclear Envelope Protein, Apq12p, and the Kinetochore in Saccharomyces
cerevisiae. Genetics.

[4] Niu W., Li Z., Zhan W., Iyer V. R., Marcotte E. M. (2008). Mechanisms
of Cell
Cycle Control Revealed by a Systematic and Quantitative Overexpression
Screen in S. cerevisiae. PLOS Genetics.

[5] Bender A., Pringle J. R. (1991). Use of a Screen
for Synthetic Lethal and Multicopy
Suppressee Mutants To Identify Two New Genes Involved in Morphogenesis
in Saccharomyces cerevisiae. Mol. Cell. Biol..

[6] Caspeta L. (2014). Biofuels. Altered sterol composition renders yeast
thermotolerant. Science.

[7] Lam F. H., Ghaderi A., Fink G. R., Stephanopoulos G. B. (2014). Engineering
alcohol tolerance in yeast. Science.

[8] Peter J. (2018). Genome evolution across 1,011 Saccharomyces cerevisiae isolates. Nature.

[9] Ihmels J. (2005). Rewiring of the yeast
transcriptional network through the evolution
of motif usage. Science.

[10] Avalos J. L., Fink G. R., Stephanopoulos G. (2013). Compartmentalization of metabolic
pathways in yeast mitochondria improves the production of branched-chain
alcohols. Nat. Biotechnol..

[11] Blazeck J., Garg R., Reed B., Alper H. S. (2012). Controlling promoter
strength and regulation in Saccharomyces cerevisiae using synthetic
hybrid promoters. Biotechnol. Bioeng..

[12] Nevoigt E. (2006). Engineering of promoter replacement cassettes for fine-tuning of
gene expression in Saccharomyces cerevisiae. Appl. Environ. Microbiol..

[13] Bro C., Regenberg B., Förster J., Nielsen J. (2006). In silico aided metabolic
engineering of Saccharomyces cerevisiae for improved bioethanol production. Metab. Eng..

[14] Schwartz C. (2019). Validating genome-wide
CRISPR-Cas9 function improves screening in
the oleaginous yeast Yarrowia lipolytica. Metab.
Eng..

[15] Schwartz C. M., Hussain M. S., Blenner M., Wheeldon I. (2016). Synthetic RNA Polymerase
III Promoters Facilitate High-Efficiency CRISPR–Cas9-Mediated
Genome Editing in Yarrowia lipolytica. ACS Synth.
Biol..

[16] Gilbert L. A. (2013). CRISPR-mediated modular
RNA-guided regulation of transcription in
eukaryotes. Cell.

[17] Gabaldón T. (2013). Comparative genomics of emerging pathogens
in the Candida glabrata
clade. BMC Genomics.

[18] Butler G. (2009). Evolution of pathogenicity and sexual reproduction in eight Candida
genomes. Nature.

[19] Dujon B. (2004). Genome evolution in yeasts. Nature.

[20] Guan Y., Dunham M. J., Troyanskaya O. G., Caudy A. A. (2013). Comparative gene
expression between two yeast species. BMC Genomics.

[21] Bao Z. (2015). Homology-Integrated CRISPR–Cas (HI-CRISPR) System for One-Step
Multigene Disruption in Saccharomyces cerevisiae. ACS Synth. Biol..

[22] Mans R. (2015). CRISPR/Cas9: a molecular
Swiss army knife for simultaneous introduction
of multiple genetic modifications in Saccharomyces cerevisiae. FEMS Yeast Res..

[23] Anzalone A. V. (2019). Search-and-replace genome
editing without double-strand breaks or
donor DNA. Nature.

[24] DiCarlo J. E. (2013). Genome engineering in Saccharomyces cerevisiae
using CRISPR-Cas systems. Nucleic Acids Res..

[25] Cámara E., Lenitz I., Nygård Y. (2020). A CRISPR activation and interference
toolkit for industrial Saccharomyces cerevisiae strain KE6–12. Sci. Rep..

[26] Jensen E. D., Ferreira R., Jakočiu̅nas T., Arsovska D., Zhang J., Ding L., Smith J. D., David F., Nielsen J., Jensen M. K. (2017). Transcriptional
reprogramming
in yeast using dCas9 and combinatorial gRNA strategies. Microb. Cell Factories.

[27] Dong C. (2020). A Single Cas9-VPR Nuclease
for Simultaneous Gene Activation, Repression,
and Editing in Saccharomyces cerevisiae. ACS
Synth. Biol..

[28] Ge H., Marchisio M. A., Aptamers R., Ribozymes in S. (2021). Cerevisiae
Synthetic Biology. Life (Basel).

[29] Ma H. (2016). Multiplexed labeling of genomic loci with dCas9 and engineered sgRNAs
using CRISPRainbow. Nat. Biotechnol..

[30] Truong V. A. (2019). CRISPRai for simultaneous gene activation and inhibition to promote
stem cell chondrogenesis and calvarial bone regeneration. Nucleic Acids Res..

[31] Zalatan J. G. (2015). Engineering Complex
Synthetic Transcriptional Programs with CRISPR
RNA Scaffolds. Cell.

[32] Qi X. (2012). Retrotransposon profiling of RNA polymerase III initiation
sites. Genome Res..

[33] Guffanti E. (2006). A minimal promoter for TFIIIC-dependent in vitro transcription of
snoRNA and tRNA genes by RNA polymerase III. J. Biol. Chem..

[34] Marck C. (2006). The RNA polymerase III-dependent
family of genes in hemiascomycetes:
comparative RNomics, decoding strategies, transcription and evolutionary
implications. Nucleic Acids Res..

[35] Ma H. (2014). Pol III Promoters to Express Small RNAs: Delineation of Transcription
Initiation. Mol. Ther Nucleic Acids.

[36] Canella D., Praz V., Reina J. H., Cousin P., Hernandez N. (2010). Defining the
RNA polymerase III transcriptome: Genome-wide localization of the
RNA polymerase III transcription machinery in human cells. Genome Res..

[37] Schramm L., Pendergrast P. S., Sun Y., Hernandez N. (2000). Different
human TFIIIB activities direct RNA polymerase III transcription from
TATA-containing and TATA-less promoters. Genes
Dev..

[38] Dieci G. (2006). Distinct modes of TATA
box utilization by the RNA polymerase III
transcription machineries from budding yeast and higher plants. Gene.

[39] Moqtaderi Z., Struhl K. (2004). Genome-wide occupancy
profile of the RNA polymerase
III machinery in Saccharomyces cerevisiae reveals loci with incomplete
transcription complexes. Mol. Cell. Biol..

[40] Oler A. J. (2010). Human RNA polymerase
III transcriptomes and relationships to Pol
II promoter chromatin and enhancer-binding factors. Nat. Struct. Mol. Biol..

[41] Zecherle G. N., Whelen S., Hall B. D. (1996). Purines
are required at the 5′
ends of newly initiated RNAs for optimal RNA polymerase III gene expression. Mol. Cell. Biol..

[42] Orioli A., Pascali C., Pagano A., Teichmann M., Dieci G. (2012). RNA polymerase III transcription control elements: Themes and variations. Gene.

[43] Mylona A. (2006). Structure of the tau60/Delta
tau91 subcomplex of yeast transcription
factor IIIC: insights into preinitiation complex assembly. Mol. Cell.

[44] Ryan O. W., Cate J. H. D. (2014). Multiplex engineering
of industrial yeast genomes using
CRISPRm. Methods Enzymol..

[45] Hassing E.-J., de Groot P. A., Marquenie V. R., Pronk J. T., Daran J.-M. G. (2019). Connecting
central carbon and aromatic amino acid metabolisms to improve de novo
2-phenylethanol production in Saccharomyces cerevisiae. Metab. Eng..

[46] Deaner M., Alper H. S. (2017). Systematic testing
of enzyme perturbation sensitivities
via graded dCas9 modulation in Saccharomyces cerevisiae. Metab. Eng..

[47] Ryan O. W., Skerker J. M., Maurer M. J., Li X., Tsai J. C., Poddar S., Lee M. E., DeLoache W., Dueber J. E., Arkin A. P. (2014). Selection of chromosomal
DNA libraries using
a multiplex CRISPR system. eLife.

[48] Papapetridis I. (2018). Laboratory evolution for forced glucose-xylose co-consumption enables
identification of mutations that improve mixed-sugar fermentation
by xylose-fermenting Saccharomyces cerevisiae. FEMS Yeast Res..

[49] Zhou P. (2019). Directed Coevolution
of β-Carotene Ketolase and Hydroxylase
and Its Application in Temperature-Regulated Biosynthesis of Astaxanthin. J. Agric. Food Chem..

[50] Li Q., Feng P., Tang H., Lu F., Mou B., Zhao L., Li N., Yang Y., Fu C., Long W. (2023). Genome-wide
identification of resistance genes and
cellular analysis of key gene knockout strain under 5-hydroxymethylfurfural
stress in Saccharomyces cerevisiae. BMC Microbiology.

[51] Bäckström A., Yudovich D., Žemaitis K., Nilsén Falck L., Subramaniam A., Larsson J. (2022). Combinatorial gene
targeting in primary
human hematopoietic stem and progenitor cells. Sci. Rep..

[52] Wieczorke R. (1999). Concurrent knock-out
of at least 20 transporter genes is required
to block uptake of hexoses in Saccharomyces cerevisiae. FEBS Lett..

[53] Liao H., Li Q., Chen Y., Tang J., Mou B., Lu F., Feng P., Li W., Li J., Fu C. (2024). Genome-wide identification
of resistance genes and response mechanism
analysis of key gene knockout strain to catechol in Saccharomyces
cerevisiae. Front. Microbiol..

[54] Winzeler E. A. (1999). Functional characterization of the S. cerevisiae
genome by gene deletion
and parallel analysis. Science.

[55] Norman T. M. (2019). Exploring genetic interaction
manifolds constructed from rich single-cell
phenotypes. Science.

[56] Horlbeck M. A. (2018). Mapping the Genetic Landscape of Human Cells. Cell.

[57] Cedras G., Kroukamp H., Van Zyl W. H., Den Haan R. (2020). The in vivo detection
and measurement of the unfolded protein response in recombinant cellulase
producing Saccharomyces cerevisiae strains. Biotechnol. Appl. Biochem..

[58] Chen H., Zhu C., Zhu M., Xiong J., Ma H., Zhuo M., Li S. (2019). High production
of valencene in Saccharomyces cerevisiae through
metabolic engineering. Microb. Cell Factories.

[59] Maury J., Kannan S., Jensen N. B., Öberg F. K., Kildegaard K. R., Forster J., Nielsen J., Workman C. T., Borodina I. (2018). Glucose-Dependent Promoters for Dynamic Regulation
of Metabolic Pathways. Front. Bioeng. Biotechnol..

[60] Promdonkoy P., Sornlek W., Preechakul T., Tanapongpipat S., Runguphan W. (2022). Metabolic Engineering of Saccharomyces
cerevisiae for
Production of Fragrant Terpenoids from Agarwood and Sandalwood. Fermentation.

[61] Sarkar D., Paira S., Das B. (2018). Nuclear mRNA
degradation tunes the
gain of the unfolded protein response in Saccharomyces cerevisiae. Nucleic Acids Res..

[62] Williams T. C., Nielsen L. K., Vickers C. E. (2013). Engineered Quorum Sensing Using Pheromone-Mediated
Cell-to-Cell Communication in Saccharomyces cerevisiae. ACS Synth. Biol..

[63] Yang X. (2021). Quorum sensing-mediated
protein degradation for dynamic metabolic
pathway control in *Saccharomyces cerevisiae*. Metab. Eng..

[64] Zhang J. (2016). Engineering an NADPH/NADP+
Redox Biosensor in Yeast. ACS Synth. Biol..

[65] Zhao E. M. (2018). Optogenetic regulation
of engineered cellular metabolism for microbial
chemical production. Nature.

[66] Zhou P. (2018). Development of a temperature-responsive yeast cell factory using
engineered Gal4 as a protein switch. Biotechnol.
Bioeng..

[67] Dabirian Y. (2019). Expanding the Dynamic
Range of a Transcription Factor-Based Biosensor
in Saccharomyces cerevisiae. ACS Synth. Biol..

[68] Zhou P., Fang X., Xu N., Yao Z., Xie W., Ye L. (2021). Development of a Highly Efficient
Copper-Inducible GAL Regulation
System (CuIGR) in Saccharomyces cerevisiae. ACS Synth. Biol..

[69] Davydenko S. (2020). Proteomics Answers Which Yeast Genes Are Specific for Baking, Brewing,
and Ethanol Production. Bioengineering.

[70] Jansen M. L. A. (2017). Saccharomyces cerevisiae strains for second-generation
ethanol production: from academic exploration to industrial implementation. FEMS Yeast Res..

[71] Molina-Espeja P. (2020). Next Generation
Winemakers: Genetic Engineering in Saccharomyces cerevisiae for Trendy
Challenges. Bioengineering.

[72] Ruta L. L., Farcasanu I. C. (2021). Saccharomyces
cerevisiae Concentrates Subtoxic Copper
onto Cell Wall from Solid Media Containing Reducing Sugars as Carbon
Source. Bioengineering.

[73] Sun M.-L., Shi T.-Q., Lin L., Ledesma-Amaro R., Ji X.-J. (2022). Advancing *Yarrowia lipolytica* as a superior biomanufacturing
platform by tuning gene expression using promoter engineering. Bioresour. Technol..

[74] Heckl D. (2014). Generation of mouse
models of myeloid malignancy with combinatorial
genetic lesions using CRISPR-Cas9 genome editing. Nat. Biotechnol..

[75] Cheng A. W. (2013). Multiplexed activation
of endogenous genes by CRISPR-on, an RNA-guided
transcriptional activator system. Cell Res..

[76] Sakuma T., Nishikawa A., Kume S., Chayama K., Yamamoto T. (2014). Multiplex
genome engineering in human cells using all-in-one CRISPR/Cas9 vector
system. Sci. Rep..

[77] Donze D., Rt K. (2001). RNA polymerase III
and RNA polymerase II promoter complexes are heterochromatin
barriers in Saccharomyces cerevisiae. EMBO journal.

[78] Bai
Flagfeldt D., Siewers V., Huang L., Nielsen J. (2009). Characterization
of chromosomal integration sites for heterologous gene expression
in Saccharomyces cerevisiae. Yeast.

[79] Lee J.-Y., Rohlman C. E., Molony L. A., Engelke D. R. (1991). Characterization
of RPR1, an essential gene encoding the RNA component of Saccharomyces
cerevisiae nuclear RNase P. MOL. CELL. BIOL..

[80] Guffanti E. (2006). Nucleosome Depletion Activates Poised RNA Polymerase
III at Unconventional
Transcription Sites in Saccharomyces cerevisiae. J. Biol. Chem..

[81] Eschenlauer J. B., Kaiser M. W., Gerlach V. L., Brow D. A. (1993). Architecture of
a yeast U6 RNA gene promoter. Mol. Cell. Biol..

[82] Harismendy O. (2003). Genome-wide location
of yeast RNA polymerase III transcription machinery. EMBO J..

[83] Waldschmidt R., Wanandi I., Seifart K. H. (1991). Identification
of transcription factors
required for the expression of mammalian U6 genes in vitro. EMBO J..

[84] Savina E. A. (2024). Structural Features
of DNA in tRNA Genes and Their Upstream Sequences. Int. J. Mol. Sci..

[85] Basehoar A. D., Zanton S. J., Pugh B. F. (2004). Identification
and distinct regulation
of yeast TATA box-containing genes. Cell.

[86] Deaner M., Mejia J., Alper H. S. (2017). Enabling
Graded and Large-Scale Multiplex
of Desired Genes Using a Dual-Mode dCas9 Activator in Saccharomyces
cerevisiae. ACS Synth. Biol..

[87] Radzisheuskaya A., Shlyueva D., Müller I., Helin K. (2016). Optimizing sgRNA position
markedly improves the efficiency of CRISPR/dCas9-mediated transcriptional
repression. Nucleic Acids Res..

[88] Auradkar A., Guichard A., Kaduwal S., Sneider M., Bier E. (2023). tgCRISPRi:
efficient gene knock-down using truncated gRNAs and catalytically
active Cas9. Nat. Commun..

[89] Li J., Kong D., Ke Y., Zeng W., Miki D. (2024). Application
of multiple sgRNAs boosts efficiency of CRISPR/Cas9-mediated gene
targeting in Arabidopsis. BMC Biology.

[90] Young E. M. (2018). Iterative algorithm-guided design of massive
strain libraries, applied
to itaconic acid production in yeast. Metab.
Eng..

[91] Anderson D. A., Voigt C. A. (2021). Competitive dCas9
binding as a mechanism for transcriptional
control. Mol. Syst. Biol..

[92] Chavez A. (2016). Comparison of Cas9 activators in multiple species. Nat. Methods.

[93] Reis A. C. (2019). Simultaneous repression
of multiple bacterial genes using nonrepetitive
extra-long sgRNA arrays. Nat. Biotechnol..

[94] Jinek M. (2012). A Programmable Dual-RNA–Guided
DNA Endonuclease in Adaptive
Bacterial Immunity. Science.

[95] Pérez-Gallardo R. V. (2013). Reactive oxygen species production induced by ethanol in Saccharomyces
cerevisiae increases because of a dysfunctional mitochondrial iron–sulfur
cluster assembly system. FEMS Yeast Res..

[96] Vergauwen B., Pauwels F., Van Beeumen J. J. (2003). Glutathione
and Catalase Provide
Overlapping Defenses for Protection against Respiration-Generated
Hydrogen Peroxide in Haemophilus influenzae. J. Bacteriol..

[97] Ng C.-H. (2008). Adaptation to hydrogen
peroxide in Saccharomyces cerevisiae: the
role of NADPH-generating systems and the SKN7 transcription factor. Free Radic Biol. Med..

[98] Tran K., Green E. M. (2019). Assessing Yeast Cell Survival Following Hydrogen Peroxide
Exposure. Bio Protoc.

[99] Song L., Ouedraogo J.-P., Kolbusz M., Nguyen T. T. M., Tsang A. (2018). Efficient
genome editing using tRNA promoter-driven CRISPR/Cas9 gRNA in Aspergillus
niger. PLoS One.

[100] Kompatscher M., Gonnella I., Erlacher M. (2025). Studying the Function
of tRNA Modifications: Experimental Challenges and Opportunities. J. Mol. Biol..

[101] Zhang Y., Wang J., Wang Z., Zhang Y., Shi S., Nielsen J., Liu Z. (2019). A gRNA-tRNA
array for CRISPR-Cas9
based rapid multiplexed genome editing in Saccharomyces cerevisiae. Nat. Commun..

[102] Daoud R., Forget L., Lang B. F. (2012). Yeast mitochondrial
RNase P, RNase Z and the RNA degradosome are part of a stable supercomplex. Nucleic Acids Res..

[103] Knapp D. J. H. F., Michaels Y. S., Jamilly M., Ferry Q. R. V., Barbosa H., Milne T. A., Fulga T. A. (2019). Decoupling tRNA
promoter and processing activities enables specific Pol-II Cas9 guide
RNA expression. Nat. Commun..

[104] Mamontov V., Martynov A., Morozova N., Bukatin A., Staroverov D. B., Lukyanov K. A., Ispolatov Y., Semenova E., Severinov K. (2022). Persistence
of plasmids targeted
by CRISPR interference in bacterial populations. Proc Natl Acad Sci U S A..

[105] Fu R., He W., Dou J., Villarreal O. D., Bedford E., Wang H., Hou C., Zhang L., Wang Y., Ma D. (2022). Systematic
decomposition
of sequence determinants governing CRISPR/Cas9 specificity. Nat. Commun..

[106] Nuñez J. K. (2021). Genome-wide programmable
transcriptional
memory by CRISPR-based epigenome editing. Cell.

[107] Ma Y., Kanakousaki K., Buttitta L. (2015). How the cell cycle impacts chromatin
architecture and influences cell fate. Front.
Genet..

[108] Mefferd A. L., Kornepati A. V. R., Bogerd H. P., Kennedy E. M., Cullen B. R. (2015). Expression of CRISPR/Cas single guide RNAs using small
tRNA promoters. RNA.

[109] Wei Y. (2017). CRISPR/Cas9 with single guide RNA expression driven
by small tRNA promoters showed reduced editing efficiency compared
to a U6 promoter. RNA.

[110] Hilton I. B. (2015). Epigenome editing by a CRISPR-Cas9-based acetyltransferase
activates genes from promoters and enhancers. Nat. Biotechnol..

[111] Tanenbaum M. E., Gilbert L. A., Qi L. S., Weissman J. S., Vale R. D. (2014). A Protein-Tagging System for Signal
Amplification in
Gene Expression and Fluorescence Imaging. Cell.

[112] Kearns N. A. (2015). Functional annotation
of native enhancers with
a Cas9–histone demethylase fusion. Nat.
Methods.

[113] Cazier A. P. (2023). A Rapid Antibody Enhancement
Platform in Saccharomyces
cerevisiae Using an Improved, Diversifying CRISPR Base Editor. ACS Synth. Biol..

[114] Portela R. M. C. (2017). Synthetic Core Promoters as Universal Parts
for Fine-Tuning Expression in Different Yeast Species. ACS Synth. Biol..

[115] Cazier A. P., Blazeck J. (2021). Advances in promoter
engineering:
Novel applications and predefined transcriptional control. Biotechnol. J..

[116] Gietz R. D., Schiestl R. H. (2007). High-efficiency
yeast transformation
using the LiAc/SS carrier DNA/PEG method. Nat.
Protoc..

[117] Hegemann J., Heick S. (2011). Delete and Repeat:
A Comprehensive
Toolkit for Sequential Gene Knockout in the Budding Yeast Saccharomyces
cerevisiae. Methods Mol. Biol..

