# Supplementary material for: RNA Polymerase III Promoters Compatible with CRISPR Gene Regulation in Saccharomyces cerevisiae
Source: ACS Synth Biol. 2025 Aug 18;14(9):3387–400. doi: 10.1021/acssynbio.5c00122 (PMC12455662; doi:10.1021/acssynbio.5c00122)
Supplement: Supplementary file 1 [file sb5c00122_si_001.pdf]

Supporting Information for:

RNA Polymerase III Promoters compatible with CRISPR Gene Regulation in *Saccharomyces cerevisiae*

Authors: Kendreze L. Holland[1,2], Ines Blancher[3], Marisa McKesey[3], Michael Silas[3], Siddhant Gandhi[3], Axum Nickerson[2], Kennedy Jackson[2], and John Blazeck[2-7*]

Author Contact Information:

[1] Department of Biomedical Engineering, Georgia Institute of Technology, Atlanta, GA 30332

[2] Bioengineering Program, Georgia Institute of Technology, Atlanta, Georgia, USA

[3] School of Chemical and Biomolecular Engineering, Georgia Institute of Technology, Atlanta, GA 30332, USA.

[4] Parker H. Petit Institute of Bioengineering and Bioscience, Georgia Institute of Technology, Atlanta, GA 30332, USA

[5] Integrated Cancer Research Center, Georgia Institute of Technology, Atlanta, GA 30332, USA

[6] Georgia Immunoengineering Consortium, Emory University and Georgia Institute of Technology, Atlanta, GA 30332, USA

[7] Winship Cancer Institute, Emory University, Atlanta, GA 30332, USA

[*] Corresponding author: john.blazeck@chbe.gatech.edu

[*] To whom correspondence should be addressed.
Tel: +1 (404)-385-4718; Email: john.blazeck@chbe.gatech.edu

Supplemental Figure S1: Transcription initiation complex interactions with Pol III promoters encoded with optimal and non-ideal, ill-defined DNA elements.

Supplemental Figure S2: Attempted alignment of the eight characterized RNA Pol III promoters.

Supplemental Figure S3: sgRNAs engineered to target different regions of pol II promoter pHTA1 and pAgTEF1 in yeast strains KH5F1 and KH4F1, respectively.

Supplemental Figure S4: Poor repression mediated by an sgRNAs in dCas9-MXI1 and dCas9 + PCP-MXI1 transcriptional regulation systems.

Supplemental Figure S5: Comparing 1X sgRNAs driven by native and non-native promoters in a CRISPRi assay.

Supplemental Figure S6: Simultaneous activation and repression of fluorescent reporter genes.

Supplemental Figure S7: Simultaneous activation and repression of fluorescent reporter genes by promoters with various architectures.

Supplemental Figure S8: Expressing crRNA and tracrRNA of a native and modified sgRNA scaffolds on separate yeast expression plasmids.

Supplemental Table S1: Sequences and Lengths of RNA Polymerase III promoters in this study

Supplemental Table S2: Efficacy of Native and Cross-Species Promoters in different CRISPR assays

Supplemental Table S3: Characterization of ill-defined Pol III DNA elements

Supplemental Table S4: Assembly Method and Primer Sequences for 1X Pol III Promoter cassettes with standard sgRNAs

Supplemental Table S5: Assembly Method and Primer Sequences for 1X Pol III Promoter cassettes and Aptamer-scaffolded sgRNAs

Supplemental Table S6: Assembly Method and Primer Sequences for 2X and 3X Pol III Promoter cassettes and Aptamer-scaffolded sgRNAs

Supplemental Table S7: Assembly Method and Primer Sequences for 1X Pol III Promoter cassettes with Separate crRNA and tracrRNAs

Supplemental Table S8: 20 bps Targeting Sequences with BsaI Overhangs and RT-qPCR primers

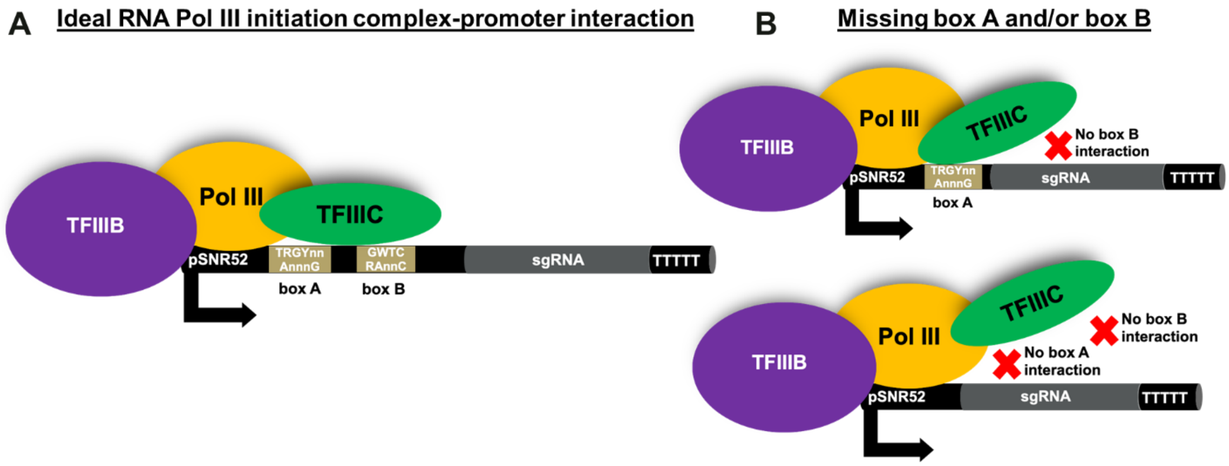

Supplemental Figure S1: Transcription initiation complex interactions with Pol III promoters encoded with optimal and non-ideal, ill-defined DNA elements. A) Proper assembly of the transcription initiation complex occurs when box A and box B elements (brown) are optimally positioned within the pSNR52 promoter (black). **B)** In the absence of either box B or both box A and box B, the complex cannot properly assemble upon the promoter due to the inability of TFIIIC to locate these regions.

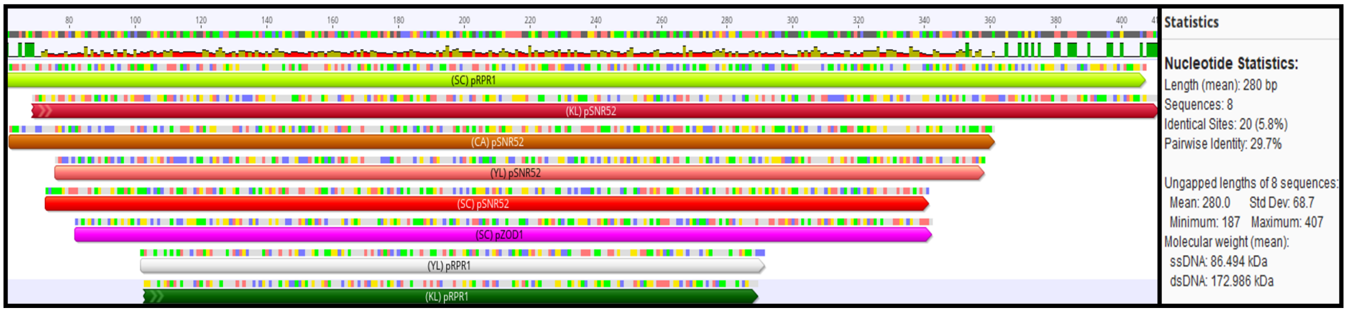

Supplemental Figure S2: Attempted alignment of the eight characterized RNA Pol III promoters. Using the 'Multiple Alignment' function in Geneious® software, DNA sequences of the Pol III promoters were aligned. Colors indicate nucleotides that differ between promoters, with only 29.7% sequence homology observed between the eight promoters. The promoters, as they appear from top to bottom, are SC pRPR1, KL pSNR52, CA pSNR52, YL pSNR52, SC pSNR52, SC pZOD1, YL pRPR1, KL pRPR1. A grey nucleotide is the most prevalent nucleotide, while the pink, yellow, purple, and green nucleotides are ‘mismatches’ from the most prevalent nucleotide, with pink = A, yellow = G, purple = C, green = T.

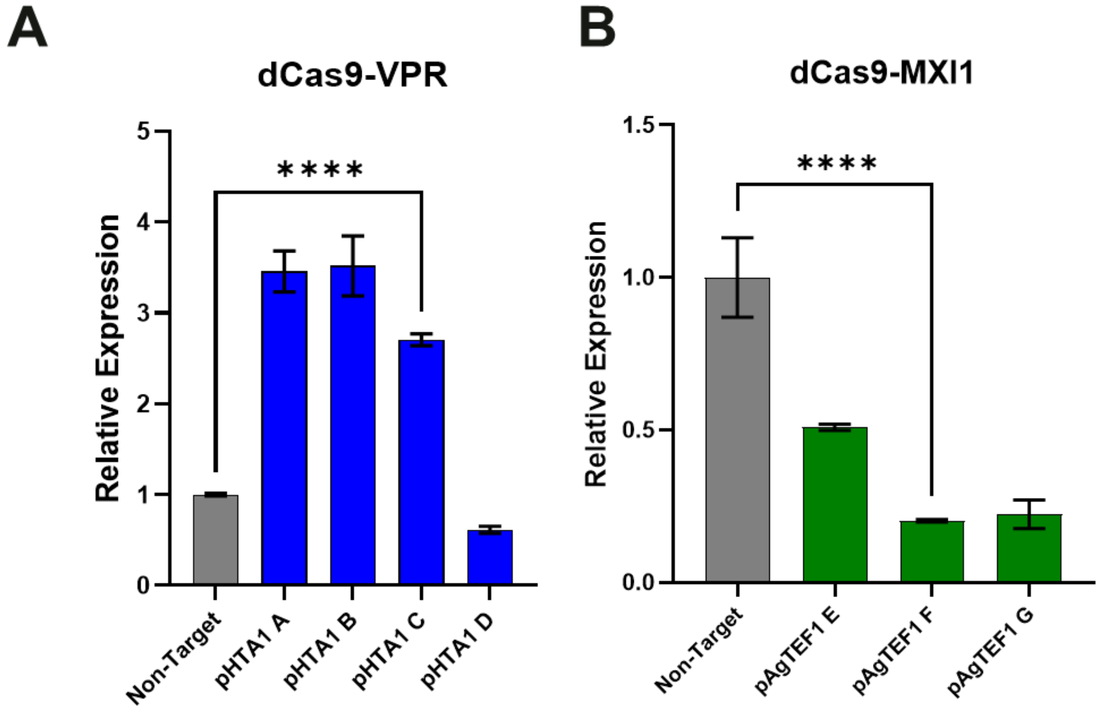

Supplemental Figure S3: sgRNAs engineered to target different regions of pol II promoter pHTA1 and pAgTEF1 in yeast strains KH5F1 and KH4F1, respectively. Spacers targeted 398 bps (HTA1 A), 311 bps (HTA1 B), 233 bps (HTA1 C), and 104 bps (HTA D) away from the start codon of the mTagBFP2 reporter in KH5F1. Spacers targeted 228 bps (AgTEF1 E), 182 bps (AgTEF1 F), and 147 bps (AgTEF1 G) away from the start codon of the yEGFP reporter in KH4F1. Error bars represent ± standard deviation of three biological replicates; ****$p<0.0001$.

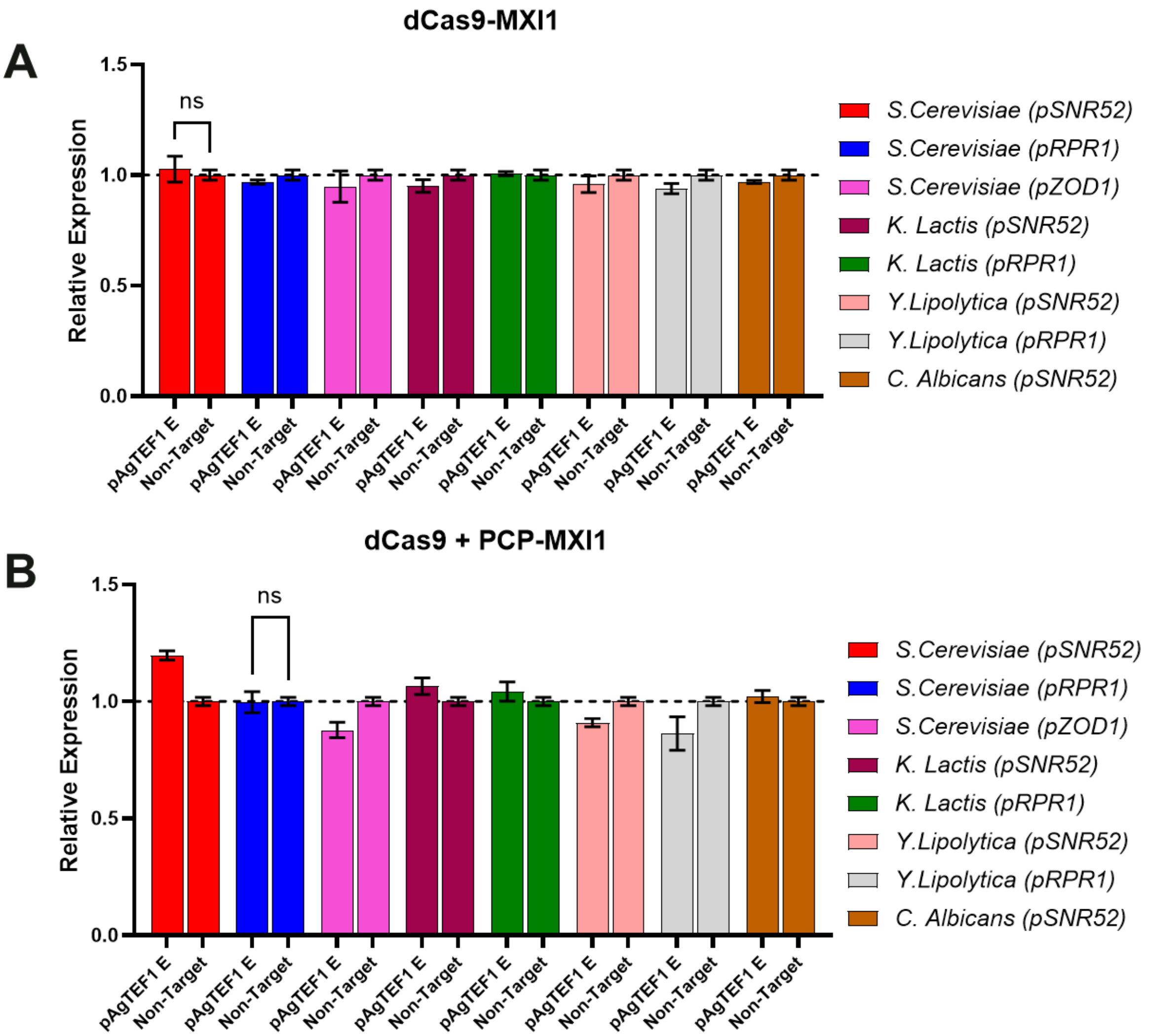

Supplemental Figure S4: Poor repression mediated by an sgRNAs in dCas9-MXI1 and dCas9 + PCP-MXI1 transcriptional regulation systems. In both yeast strains (**KH4F1** and **KH7F1**), which use different modes of transcriptional regulation, targeting the Pol II promoter, pAgTEF1, 228 bp upstream of the start codon, is not optimal for transcriptional repression of yEGFP. Error bars represent ± standard deviation of three biological replicates. ns= not significant.

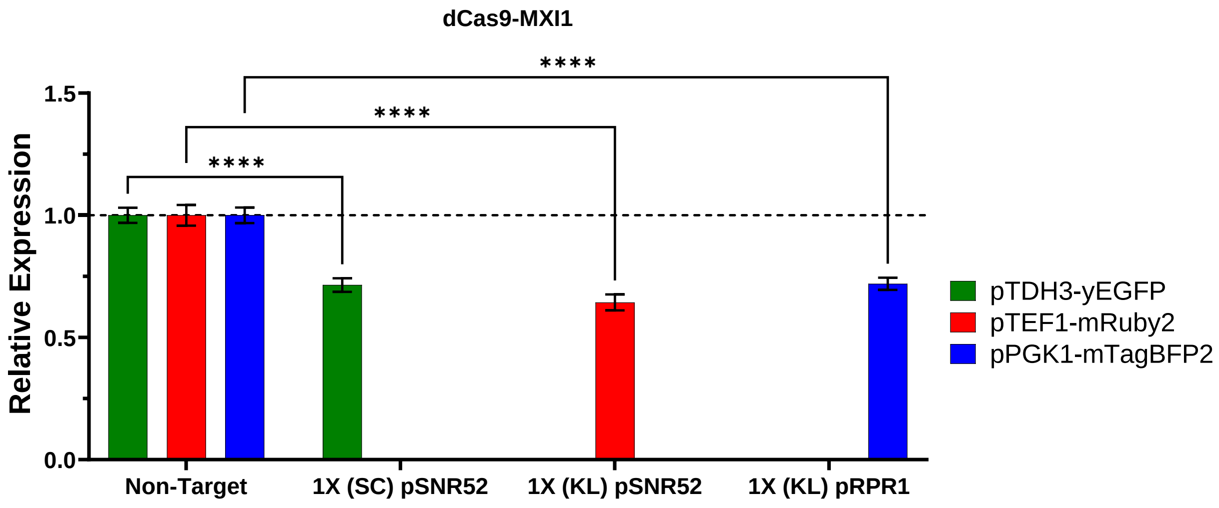

Supplemental Figure S5: Validation of individual sgRNAs used for 3X CRISPRi. Native *S. cerevisiae* pSNR52, non-native *K. lactis* pSNR52, and *K. lactis* pRPR1 promoters each drove the expression of a distinct 1X sgRNA targeting separate constitutive Pol II promoters in the three-color KH4F strain. Each promoter–sgRNA pair significantly repressed the corresponding fluorescent reporter. The error bars represent ± standard deviation of three biological replicates; ****$p<0.0001$.

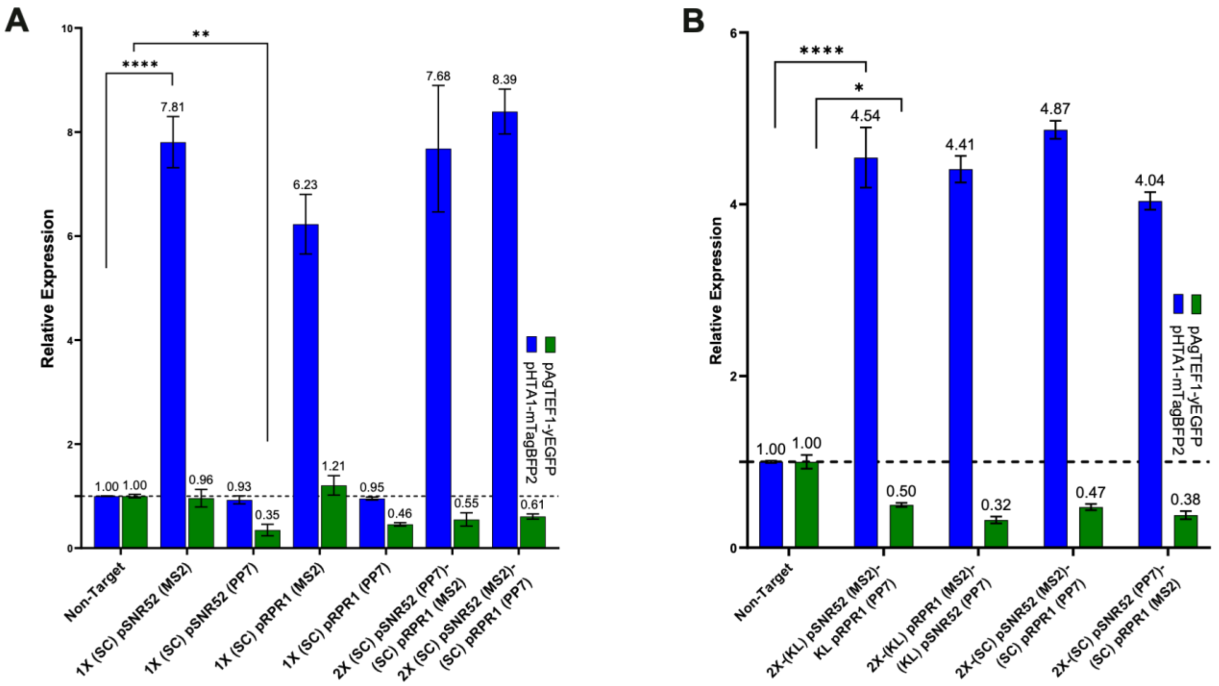

Supplemental Figure S6: Simultaneous activation and repression of fluorescent reporter genes. A) The data represent our first demonstration of simultaneous activation and repression of mTagBFP2 and yEGFP by *S. cerevisiae* Pol III promoters (i.e., p*SNR52* and p*RPR1*) assembled into a 2X cassette, as quantified by flow cytometry. Switching the positions of the aptamers in the sgRNAs within the 2X cassettes did not impact fluorescent reporter expression. **B)** This data compares the efficacy of simultaneous transcriptional activation and repression by 2X cassettes expressing Pol III promoters driving sgRNAs with aptamers from *S. cerevisiae* and *K. lactis*. Again, changing the orientation of the components in the sgRNAs, in addition to promoter rearrangement, did not lead to a significant loss in the activation or repression of the targeted fluorescent reporters. Values greater than one indicate activation of mTagBFP2, while values less than one indicate repression of yEFP. In **A** and **B,** each experiment was performed in yeast strain KH7F1, error bars represent ± standard deviation of three biological replicates; ****$p<0.0001$; **$p<0.01$; *$p<0.05$.

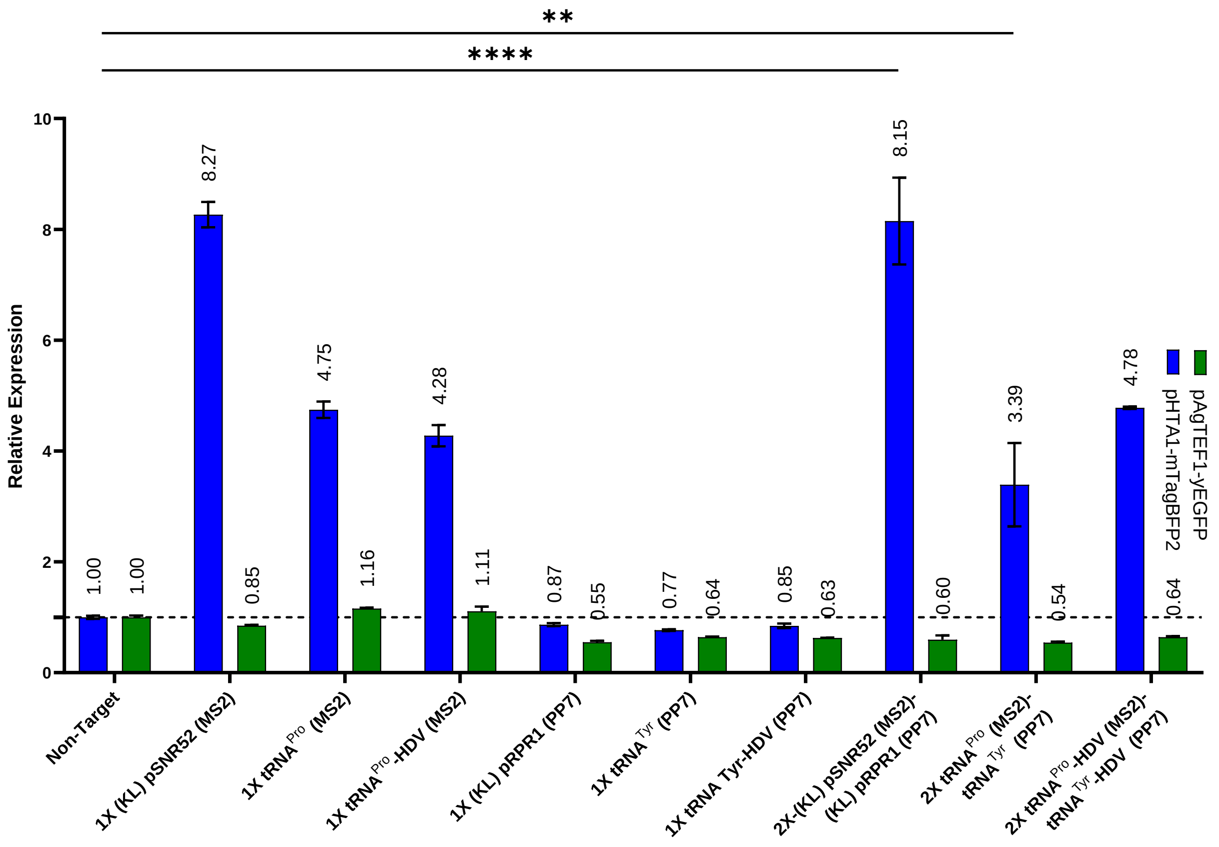

Supplemental Figure S7: Simultaneous activation and repression of fluorescent reporter genes by promoters with various architectures. KL pSNR52 and pRPR1 were compared to $tRNA^{Pro}$ and $tRNA^{Tyr}$, either with or without the addition of an HDV ribozyme, for simultaneous gene regulation. KL-constructs had improved CRISPRa activity, and addition of HDV did not impact expression control mediated by tRNA constructs. Values greater than one indicate activation of mTagBFP2, while values less than one indicate repression of yEFP. This experiment was performed in yeast strain KH7F1, error bars represent ± standard deviation of three biological replicates; ****$p<0.0001$; **$p<0.01$.

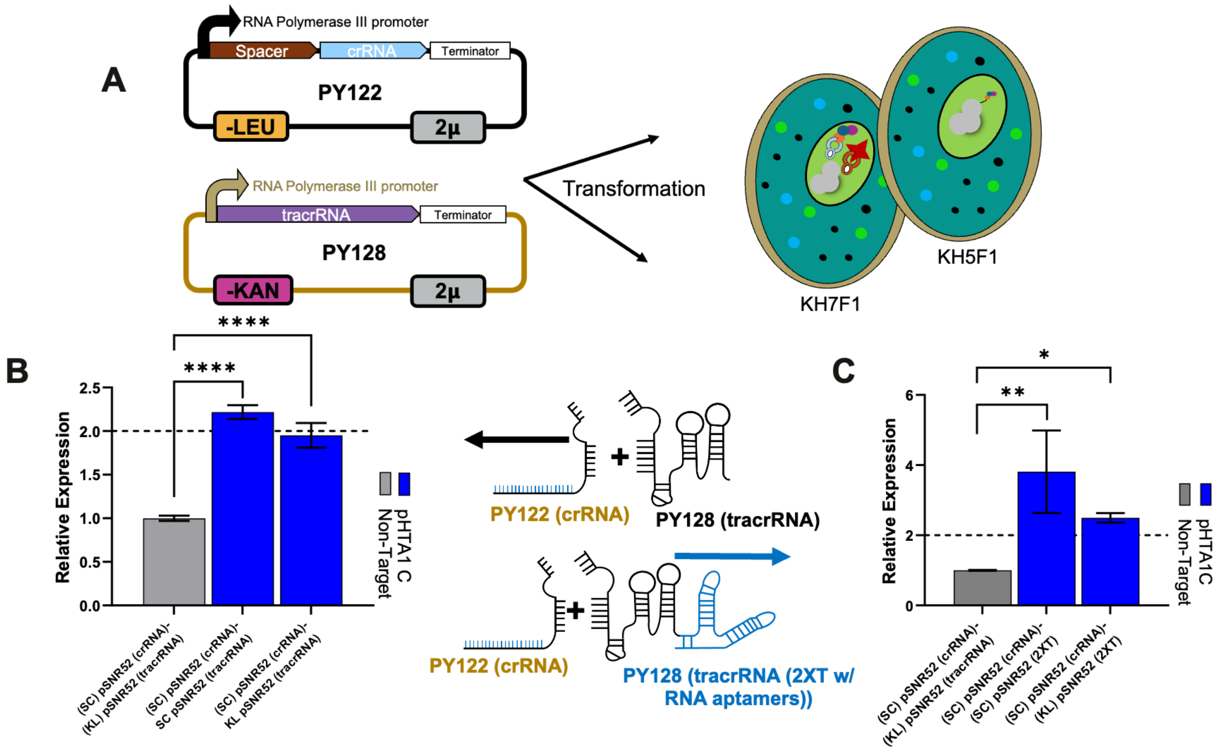

Supplemental Figure S8: Expressing separated crRNA and tracrRNA of standard and scaffolded sgRNAs on separate yeast expression plasmids. (A) Yeast expression plasmids with the configurations: PY122-Pol III Promoter-spacer(crRNA)-terminator and PY128-Pol III promoter-tracrRNA-terminator were co-transformed into two separate yeast strains expression dCas9-VPR (KH5F1) or dCas9 +MCP/VPR (KH7F1). Both yeast strains were engineered to express mTagBFP2 under the weak pHTA1 Pol II promoter. **(B-C)** In both yeast strains, the two co-transformed plasmids successfully activated the mTagBFP2 expression compared to the non-target controls. 2XT = 2X Tail; crRNA = CRISPR RNA; tracrRNA = trans-activating CRISPR RNA. Error bars represent ± standard deviation of three biological replicates; ****$p<0.0001$; **$p<0.01$; *$p<0.05$.

Supplemental Table S1: Sequences and Lengths of RNA Polymerase III promoters in this study

Promoter Name	Promoter Sequence	Species of origin	Length
SC pSNR52	TCTTTGAAAAGATAATGTATGATTATGCTTTCACTCATATTTATACAGAAACTTGATGTTTTCTTTCGAGTATATACAAGGTGATTACATGTACGTTTGAAGTACAACTCTAGATTTTGTAGTGCCCTCTTGGGCTAGCGGTAAAGGTGCGCATTTTTTCACACCCTACAATGTTCTGTTCAAAAGATTTTGGTCAAACGCTGTAGAAGTGAAAGTTGGTGCGCATGTTTCGGCGTTCGAAACTTCTCCGCAGTGAAAGATAAATGATC	*Saccharomyces cerevisiae* (SC)	269
SC pRPR1	GATCTGCCAATTGAACATAACATGGTAGTTACATATACTAGTAATATGGTTCGGCACACATTAAAAGTATAAAAACTATCTGAATTACGAATTACATATATTGGTCATAAAAATCAATCAATCATCGTGTGTTTTATATGTCTCTTATCTAAGTATAAGAATATCCATAGTTAATATTCACTTACGCTACCTTTTAACCTGTAATCATTGTCAACAGGATATGTTAACGACCCACATTGATAAACGCTAGTATTTCTTTTTCCTCTTCTTATTGGCCGGCTGTCTCTATACTCCCCTATAGTCTGTTTCTTTTCGTTTCGATTGTTTTACGTTTGAGGCCTCGTGGCGCACATGGTACGCTGTGGTGCTCGCGGCTGGGAACGAAACTCTGGGAGCTGCGATTGGCA	*Saccharomyces cerevisiae* (SC)	407
SC pZOD1	CGTTGCATTACGTGGTGTCCGGTCGCTATTTCCTGGGTTTTCTGCGGCAGATTCGCACTGCGCGCCACAGCTGCAACAACTGCAGTGTTATTTCGGGTGCCAATTTAGCGTGCTTCGATCTAACTATTCTGTAAATTCTGTAGTGACTCTTTAGGGCGAAGTACGTATTTCATGATTTAAAATCCCCGTCTTCAAATATACCGAAAAAAGAAACAAGACATTTTTTAGCAGTTTATGTACGCATTTTAAAAGCCGGGAAAT	*Saccharomyces cerevisiae* (SC)	261
SC pSCR1	CCAGGAACTTTTCATTGAAAATCTTCTGACAAATTTTCTGTGTAAAGGAAAGAAAATGGAGTATAAAATCGAAAGTTTATTCCAATTGTGCT	*Saccharomyces cerevisiae* (SC)	92
SC Extended pSCR1	TCCTTGTTATAACAACCCTCGGTATTCTTTTAACAAACTTACGATGACTATATTGTATTAGTGTCAACCTCATCGCCAGGAACTTTTCATTGAAAATCTTCTGACAAATTTTCTGTGTAAAGGAAAGAAAATGGAGTATAAAATCGAAAGTTTATTCCAATTGTGCT	*Saccharomyces cerevisiae* (SC)	167
SC pU6	GTTTCAACACAGCCTGGCATGAACAGTGGTAAAAGTATTTCGTCCACTATTTTCGGCTACTATAAATAAATGTTTTTTTCGCAACTATGT	*Saccharomyces cerevisiae* (SC)	90
SC pRNA170	GGCTATAATGGTCTGGGCTAGTCTTAGTTTGATTGAGCGACAAGAGAGATCTGAACCCAGTGTCAAACGTTACCGCTTCTTTCTCTTATATACCA	*Saccharomyces cerevisiae* (SC)	95
Mouse pU6	GATCCGACGCCGCCATCTCTAGGCCCGCGCCGGCCCCCTCGCACAGACTTGTGGGAGAAGCTCGGCTACTCCCCTGCCCCGGTTAATTTGCATATAATATTTCCTAGTAACTATAGAGGCTTAATGTGCGATAAAAGACAGATAATCTGTTCTTTTTAATACTAGCTACATTTTACATGATAGGCTTGGATTTCTATAAGAGATACAAATACTAAATTATTATTTTAAAAAACAGCACAAAAGGAAACTCACCCTAACTGTAAAGTAATTGTGTGTTTTGAGACTATAAATATCCCTTGGAGAAAAGCCTTGTT	*Mus Musculus*	314
Human pU6	GAGGGCCTATTTCCCATGATTCCTTCATATTTGCATATACGATACAAGGCTGTTAGAGAGATAATTAGAATTAATTTGACTGTAAACACAAAGATATTAGTACAAAATACGTGACGTAGAAAGTAATAATTTCTTGGGTAGTTTGCAGTTTTAAAATTATGTTTTAAAATGGACTATCATATGCTTACCGTAACTTGAAAGTATTTCGATTTCTTGGCTTTATATATCTTGTGGAAAGGAC	*Homo sapiens*	241
KL pRPR1	GGAGTTGATAATTTACATAAGTTTAATTATATTCTCGTCCTTGGCCTCGTGGATGATACTCTGAACTACGGAAATGACACCTGACACACACGTGACAGGGTCTACTACTGAATGAATTCGTAATTGGTTTGAGTCGGGTGTTGTGCCGTAGCTGGTAAAAAGGCCACGTTGCGACTTCGGTTCGAAT	*Kluyveromyces lactis* (KL)	187
KL pSCR1	GGCATGGCTGCATGAAGTATATCAGATATAGTCGTCGTGTATATTCAAAGTAAAAATATGTGTATATGTATATCCAGGTTTTTCCTCTAGCGCATAAGTATATCATATTAGAGGAGTAATCCCTAGGGCTGGAAATTGAATAAAGAAAAACCTTCACGTGATCGCCTCAATCTGCTATCATCTTCTTTTTTCCTCAAATATGTTTAAGAAAGCACCCTGTCGAA	*Kluyveromyces lactis* (KL)	224
KL pSNR52	AATGAGGGAAAGAGGGAAATCGTTATGAACGAAACCAGGGTAACAATGAGCGATGACCACGTCTCCCAAGGTTGTCGTTTTAGGATATTGAACCAGGAAATGCTAGAGGCTGGAATGGCGTAATGGAACCGTGCACTGTGGTCGAAGGGGATGTTTATTCTTGCAGTTTGGGAAACGTCTCGTGATAGGCTCTCACAACATAACGGGCAAACGCCTCTAAGAAATACCTTGCTTCTTATTAACCCCCAGTAGCTTAGTGGGCGGACCAGGCGGTCTGCGCGAATTGCCAGAAAACCGAAGTTGTGACAAATAAAAAAAAGTTACTTTCTCGGCAGTTCGAATC	*Kluyveromyces lactis* (KL)	343
YL pSNR52	GACCCCGTCTTCAATTACACTTCCCAACTGGGAACACCCCTCTTTATCGACCCATTTTAGGTAATTTACCCTAGCCCATTGTCTCCATAAGGAATATTACCCTAACCCACAGTCCAGGGTGCCCAGGTCCTTCTTTGGCCAAATTTTAACTTCGGTCCTATGGCACAGCGGTAGCGCGTGAGATTGCAAATCTTAAGGTCCCGAGTTCGAATCTCGGTGGGACCTAGTTATTTTTGATAGATAATTTCGTGATGATTAGAAACTTAACGCAAAATAATGACGT	*Yarrowia lipolytica* (YL)	283
YL pRPR1	TATATATTTTTTATTCTAATGATTCATTACAGCTATATACCTAGTAAGCCGGGTTATTGGCGTTCAATAAATCATACACTTCTGAATCTTTGATTACAGTCATTCCTGACGACTGTCCGATGAAACGGCCTAAAAAAGTTAAAATGTCGGGAAATTTAGCTCTGGCCTAACGGTCAGACTGCAGTTACCC	*Yarrowia lipolytica* (YL)	190
AG pRPR1	ACCCTTCCAATAAACGTCGACATGCACTGAGACACTATTTCCCGTTTTATACATACTGACATTATCTTTGAGTCACGTGACCGAGCTACGTAGCTTTCCGGCGCCAGTCAAGTGTCAGAGGACTCTCCAATGCTCGAATCCAGTCGCTAGAGGCCGCACGCCCAAGGCTCCGAGCATATGCTCGCGTAGCAGGCGAGTCCAAGAAATACGCCTGTCTAGAATAGGACGCAGCAGGGGGGGCGCACAGCGCCCTTTGAGGTCCTGCAGTTCTGGTTCAACGTAGA	*Ashbya gossypii* (AG)	283
AG pSCR1	CCTACTGTGCGTCACGTCTGTATCTAGTGCCAACCGCCGCCTCTCAGCTAGTGTCCACCTTCCAGGATTGGATACCACAGCGCGACTGGTCCGATGTGATGCTCCTGTTCCTTACGCTGCGAGTGCGCTGATCCGTTATCGTTTCCTGCCAGCTATATGCCTTCGTGGGCGCCTCTCTTTTAGCTGTGACGGCCTTTGGCCGGAAGGGACATTAGCAATCCCGGCTTGGTGAGGCGCTCCCGCGTTCGAACCTGCGGCCGGGCATGGTTGCGGAAAAGCGAGGTTGCCTAGCTCCTCGTGCGCCATGATACCACACTGGT	*Ashbya gossypii* (AG)	320
AG pSNR52	GTGCTGGGTTGCGGCGTGCGGAGGAAAACACACCATATAAATATCAAAAATATCTATGTGGAGCTAGTAAATAAATAAAGAAGGGAACGGGGCATGAGTCCTGTCCTGTTCGGTAACAGATCGTTGTGTGGTTGGCCGGCAAATTGCTTTTTCGGGCGCGATAGTGTTGGCGGCGAAAAATTTTCAGTG	*Ashbya gossypii* (AG)	189
CA pRPR1	CCCTGTTCAGGCGGTCAATTAGTCAAGAAATAACGGATAAAAGAGAGGAATACTTTCTGTGATTGGGTACCATATACTGTTCTATAACCAGGAGGGGAAAAACTGTAAAATTTCTCCTCAATTTGTTGAATTGCTTGCAGTACCACTATTATGCATCTATAACACATAAACTTCATGCATTTCAAATTACATAGATCAGGCACCTGAATTACTTTTACTTGCATGGCCGCATATCCTAAAACTGACCAGGAGAATAAAAAAAAGCTATAGTCAAAACTGTCGAGCTAGACCGAACTGAAGTGTTTATCAATATTGAAAGACCAATTCATAGAGTATCAGTTACACCAAGAAGCAACTAAAACCAATATTCATTGTAAAACTTCTGTGCAATTTCGCACGACCGTATCAGCATACAAAATAACTCCTGCGTATCAAAGATTGACTATTCCAAGCTTTTGGCGTAATCGCAC	*Candida albicans* (CA)	470
CA pSCR1	CTTTGGAGAAAGTTATTTTTATTCAAACCTAGATATTTGGTTCAGCTAGGAAGTTTCATTACGATTTGGTAGAAAATTTAACCTAAAAAGATTATCACCTCAAACCTTCAATAATTGTTTCTAAAAAAATCCTCTAAAACAAGCTTGTGACCTCAAGACCAATCACAATTTTATGCATGAAAGAGTATTAAGCAGATGAACATTATTGAGAAAATATATATCTCAAG	*Candida albicans* (CA)	227
CA pSNR52	ATTACGTACCAAGAAGCATCTAATCAACTCCCAGATCACCACTATACATTTAAAAGTCATTGGTCAATAACTATACTCGAGTATTGCCTCATCAAAGAAACAATCAAATATTATAGATACTCACTCCATCACGTGATAATTTCACTGGTATGGAAAAGTGGAAAATTTTATAAAAAAAAAATTTGATGCCTTTGGCATAGCTGAAACTTCGGCCCAATAGGATTGGAGAATATGTTTTCGCAGCGTTCTTACAATTAAATTGTGGTGGAAGTTCGAGACTTGCGTAAACTATTTTTAATTT	*Candida albicans* (CA)	300
SC pSNR52-HDV	TCTTTGAAAAGATAATGTATGATTATGCTTTCACTCATATTTATACAGAAACTTGATGTTTTCTTTCGAGTATATACAAGGTGATTACATGTACGTTTGAAGTACAACTCTAGATTTTGTAGTGCCCTCTTGGGCTAGCGGTAAAGGTGCGCATTTTTTCACACCCTACAATGTTCTGTTCAAAAGATTTTGGTCAAACGCTGTAGAAGTGAAAGTTGGTGCGCATGTTTCGGCGTTCGAAACTTCTCCGCAGTGAAAGATAAATGATCGATGGCCGGCATGGTCCCAGCCTCCTCGCTGGCGCCGGCTGGGCAACACCTTCGGGTGGCGAATGGGACTTT	*Saccharomyces cerevisiae* (SC)	341
tRNA^{Pro}-HDV	TCCGTAGTCTAAATGAGTTACGTACGAAAGGGAGCCAAAAGCAATCTGACCAATTTGTATATATATACATCTATCCGAAAAGGAAGACATCAATTAGTACGGGCGTGTGGTCTAGTGGTATGATTCTCGCTTTGGGCGACTTCCTGATTAAACAGGAAGACAAAGCATGCGAGAGGCCCTGGGTTCAATTCCCAGCTCGCCCCGATGGCCGGCATGGTCCCAGCCTCCTCGCTGGCGCCGGCTGGGCAACACCTTCGGGTGGCGAATGGGACTTT	*Saccharomyces cerevisiae* (SC)	275
tRNA^{Tyr}-HDV	GTATACTCTTTCTTCAACAATTAAATACTCTCGGTAGCCAAGTTGGTTTAAGGCGCAAGACTGTAATTTATCACTACGAAATCTTGAGATCGGGCGTTCGACTCGCCCCCGGGAGAGATGGCCGGCATGGTCCCAGCCTCCTCGCTGGCGCCGGCTGGGCAACACCTTCGGGTGGCGAATGGGACTTT	*Saccharomyces cerevisiae* (SC)	188
KL pRPR1-HDV	AATGAGGGAAAGAGGGAAATCGTTATGAACGAAACCAGGGTAACAATGAGCGATGACCACGTCTCCCAAGGTTGTCGTTTTAGGATATTGAACCAGGAAATGCTAGAGGCTGGAATGGCGTAATGGAACCGTGCACTGTGGTCGAAGGGGATGTTTATTCTTGCAGTTTGGGAAACGTCTCGTGATAGGCTCTCACAACATAACGGGCAAACGCCTCTAAGAAATACCTTGCTTCTTATTAACCCCCAGTAGCTTAGTGGGCGGACCAGGCGGTCTGCGCGAATTGCCAGAAAACCGAAGTTGTGACAAATAAAAAAAAGTTACTTTCTCGGCAGTTCGAATCGATGGCCGGCATGGTCCCAGCCTCCTCGCTGGCGCCGGCTGGGCAACACCTTCGGGTGGCGAATGGGACTTT	*Kluyveromyces lactis* (KL)	415
KL pSNR52-HDV	GGAGTTGATAATTTACATAAGTTTAATTATATTCTCGTCCTTGGCCTCGTGGATGATACTCTGAACTACGGAAATGACACCTGACACACACGTGACAGGGTCTACTACTGAATGAATTCGTAATTGGTTTGAGTCGGGTGTTGTGCCGTAGCTGGTAAAAAGGCCACGTTGCGACTTCGGTTCGAATGATGGCCGGCATGGTCCCAGCCTCCTCGCTGGCGCCGGCTGGGCAACACCTTCGGGTGGCGAATGGGACTTT	*Kluyveromyces lactis* (KL)	259

Supplemental Table S2: Attempted alignment of the eight characterized RNA Pol III promoters.

Promoter Name	Species of origin	CRISPRa (dCas9-VPR)	CRISPRi (dCas9-MXI1)	CRISPR w/ RNA aptamers (MS2 1,3-MCP-VPR)	CRISPR w/ RNA aptamers (PP7 1,3-PCP-MXI1)	"+" Represents the number of sgRNAs that were significantly greater than the non-target control for figures 2-3
SC pSNR52	*(SC) Saccharomyces cereviriae*	+++	++	+++	++	"-" Indicates sgRNA(s) that did not show a significant difference compared to the non-targeting control.
SC pRPR1	*(SC) Saccharomyces cereviriae*	+++	++	+++	++	
SC pZOD1	*(SC) Saccharomyces cereviriae*	+	+	+++	+	
SC pSCR1	*(SC) Saccharomyces cereviriae*	-	-	-	-	
SC Extended pSCR1	*(SC) Saccharomyces cereviriae*	-	-	-	-	
SC pU6	*(SC) Saccharomyces cereviriae*	-	-	-	-	
SC pRNA170	*(SC) Saccharomyces cereviriae*	-	-	-	-	
YL pSNR52	*(YL) Yarrowia lipolytica*	-	++	+++	++	
YL pRPR1	*(YL) Yarrowia lipolytica*	-	-	+++	++	
AG pRPR1	*(AG) Ashbya gossypii*	-	-	-	-	
AG pSCR1	*(AG) Ashbya gossypii*	-	-	-	-	
AG pSNR52	*(AG) Ashbya gossypii*	-	-	-	-	
CA pRPR1	*(CA) Candida albicans*	-	-	-	-	
CA pSCR1	*(CA) Candida albicans*	-	-	-	-	
CA pSNR52	*(CA) Candida albicans*	-	-	+++	-	
KL pRPR1	*(KL) Kluyveromyces lactis*	++	++	+++	++	
KL pSCR1	*(KL) Kluyveromyces lactis*	-	-	-	-	
KL pSNR52	*(KL) Kluyveromyces lactis*	++	++	+++	++	
Human pU6	Mammalian	-	-	-	-	
Mouse pU6	Mammalian	-	-	-	-	

Promoter Name	Species of origin	CRISPRa (dCas9-VPR)	CRISPRi (dCas9-MXI1)	Multiplexing with CRISPRi (dCas9-MXI1)	CRISPR w/ RNA aptamers (MS2 1,3-MCP-VPR)	CRISPR w/ RNA aptamers (PP7 1,3-PCP-MXI1)
SC pSNR52	*(SC) Saccharomyces cereviriae*	+	+	++	++	++
SC pSNR52-HDV	*(SC) Saccharomyces cereviriae*	+	+	++	++	++
tRNA^{Pro}	*(SC) Saccharomyces cereviriae*	+	-	++	++	++
tRNA^{Pro} -HDV	*(SC) Saccharomyces cereviriae*	+	-	++	++	++
tRNA^{Tyr}	*(SC) Saccharomyces cereviriae*	+	-	++	++	++
tRNA^{Tyr} -HDV	*(SC) Saccharomyces cereviriae*	+	-	++	++	++
KL pRPR1	*(KL) Kluyveromyces lactis*	+	+	++	++	++
KL pRPR1-HDV	*(KL) Kluyveromyces lactis*	+	-	++	++	++
KL pSNR52	*(KL) Kluyveromyces lactis*	+	+	++	++	++
KL pSNR52-HDV	*(KL) Kluyveromyces lactis*	+	+	++	++	++

Supplemental Table S3: Characterization of ill-defined Pol III DNA elements

Promoter Name	TATAWAWR	Box A (TRGYnnARBGG)	Box B (GWTCRAnnC)	Length between Box A and B	Mismatches in Box A	Mismatches in Box B	TATA box (TATAWAWR) position relative to Box A and B
SC pSNR52	Yes	TTGGGCTAGCGGTAAAGG	CGTTCGAAAC	86 bps	1 (Guffanti et al. 2006)	0 (Guffanti et al. 2006)	Upstream of Box A
SC pRPR1	Yes	TGGCGCACATG	GGAACGAAACT	23 bps	Lee *et al.* 1990	Lee et al. 1990	Upstream of Box A
SC pZOD1	Yes	CGGCAGATTCG	N/A	N/A	1	N/A	Downstream of Box A
SC pSCR1	Yes	TATTCCAATTG	N/A	N/A	2	N/A	Upstream of Box A
SC Extended pSCR1	Yes	TATTCCAATTG	N/A	N/A	1	N/A	Upstream of Box A
SC pU6	Yes	TGGTAAAAGTA	N/A	N/A	2	N/A	Downstream of Box A/B
SC pRNA170	Yes	N/A	N/A	N/A	N/A	N/A	N/A
Human pU6	Yes	TTTTAAAATGG	TTTCGATTT	31	2	2	Downstream of Box A/B
Mouse pU6	Yes	AGGCTTAATGT	TTTTAATAC	26	2	2	Downstream of Box A/B
YL pSNR52	Yes (two mismatches)	TGGCACAGCGG	GTTCGAATC	33	0	0	Upstream of Box A
YL pRPR1	YES	TGGCCTAACGG	N/A (in gene)	N/A	0	N/A	Upstream of Box A
AG pRPR1	Yes	CAGCAGGGGGG	GTTCAACGT	32	2	1	Upstream of Box A
AG pSNR52	Yes	TGGTTGGCCGG	GTTGGCGGC	34	2	2	Upstream of Box A
AG pSCR1	Yes (two mismatches)	TGCCTTCGTGG	N/A	N/A	2	N/A	Upstream of Box A
CA pRPR1	Yes	TACTTGCATGG	GACCAATTC	92	2	1	Upstream of Box A
CA pSCR1	Yes	TGGTTCAGCTA	TATCACCTC	44	2	2	Downstream of Box A/B
CA pSNR52	Yes	TGGCATAGCTG	GTTCGAGAC	67	1	0	Upstream of Box A
KL pRPR1	Yes	TGGTAAAAAGG	GTTCGAATC	16	1	0	Upstream of Box A
KL pSCR1	Yes	TATTAGAGGAG	GATCGCCTC	43	2	1	Upstream of Box A
KL pSNR52	Yes (two mismatches)	TAGCTTAGTGG	GTTCGAATC	74	0	0	Upstream of Box A
tRNA^{Pro}	Yes	TGGTCTAGTGG	GTTCAATTC	64	0	0	Upstream of Box A
tRNA^{Tyr}	Yes (two mismatches)	TAGCCAAGTTG	GTTCGACTC	50	1	0	Upstream of Box A

Supplemental Table S4: Assembly Method and Primer Sequences for 1X Pol III Promoter cassettes with standard sgRNAs

1X Pol III Promoter Cassette Plasmids (standard sgRNAs)	Assembly Method	Backbone	Fragments	Forward Primer	Reverse Primer
SC pSNR52	Gibson	PY122:BsaI cut; Chloramphenicol; -Leu; 2μ	pSNR52-KanR Dummy Region	KH272: 5'-GTTGATTGGAAGACCTGTGCTCTTTGAAAAGATAATGTATGATTATGCTTTC-3'	KH040: 5'-CTCTGAAACTGAGACCGAAGGAGAAAACTCACCGAGG-3'
			gRNA scaffold-tSUP4	KH041: 5'-GTTTTCTCCTTCGGTCTCAGTTTCAGAGCTAGAAATAGCAAGTTG-3'	KH285: 5'-ATACCGCTCGAAGACCTGAGGAGACATAAAAAACAAAAAAAGCACCGACTCGGTGCC-3'
SC pRPR1	Gibson	PY122:BsaI cut; Chloramphenicol; -Leu; 2μ	pRPR1	KH315: 5'-GTTGATTGGAAGACCTGTGCGATCTGCCAATTGAACATAACATGG-3'	KH497: 5'-GCGAGCGGTCTCACAAGTGCCAATCGCAGCTCC-3'
			KanR Dummy-gRNA scaffold-tSUP4	KH498: 5'-GGAGCTGCGATTGGCACTTGTGAGACCGCTCGC-3'	KH285: 5'-ATACCGCTCGAAGACCTGAGGAGACATAAAAAACAAAAAAAGCACCGACTCGGTGCC-3'
SC pZOD1	Gibson	PY122:BsaI cut; Chloramphenicol; -Leu; 2μ	pZOD1	KH428:5'-GTTGATTGGAAGACCTGTGCCGTTGCATTACGTGGTGTCC-3'	KH489:5'-GCGAGCGGTCTCACAAGATTTCCCGGCTTTTAAAATGCG-3'
			KanR Dummy-gRNA scaffold-tSUP4	KH490: 5'-CGCATTTTAAAAGCCGGGAAATCTTGTGAGACCGCTCGC-3'	KH285: 5'-ATACCGCTCGAAGACCTGAGGAGACATAAAAAACAAAAAAAGCACCGACTCGGTGCC-3'
YL pSNR52	Gibson	PY122:BsaI cut; Chloramphenicol; -Leu; 2μ	pSNR52	KH334: 5'-GTTGATTGGAAGACCTGTGCGACCCCGTCTTCAATTACACTTC-3'	KH491: 5'- GCGAGCGGTCTCACAAGACGTCATTATTTTGCGTTAAGTTTCTAATC
			KanR Dummy-gRNA scaffold-tSUP4	KH492: 5'-GATTAGAAACTTAACGCAAAATAATGACGTCTTGTGAGACCGCTCGC-3'	KH285: 5'-ATACCGCTCGAAGACCTGAGGAGACATAAAAAACAAAAAAAGCACCGACTCGGTGCC-3'
YL pRPR1	Gibson	PY122:BsaI cut; Chloramphenicol; -Leu; 2μ	pRPR1	KH479: 5'-GTGAGTTGATTGGAAGACCTGTGCTATATATTTTTTATTCTAATGATTCATTACAGCTATATACC-3'	KH495: 5'-GCGAGCGGTCTCACAAGGGGTAACTGCAGTCTGACC-3'
			KanR Dummy-gRNA scaffold-tSUP4	KH496: 5'-GGTCAGACTGCAGTTACCCCTTGTGAGACCGCTCGC-3'	KH285: 5'-ATACCGCTCGAAGACCTGAGGAGACATAAAAAACAAAAAAAGCACCGACTCGGTGCC-3'
AG pRPR1	Gibson	PY122:BsaI cut; Chloramphenicol; -Leu; 2μ	pRPR1-pHTA1-C	KH460: 5'- CAGTGAGTTGATTGGAAGACCTGTGCACCCTTCCAATAAACGTCGAC-3'	KH461: 5'-TCTGAAACCTGGCTGGCTTCGTGAACGGCAAGTCTACGTTGAACCAGAACTGC-3'
			pHTA1-C-gRNA-tSUP4	KH462: 5'-CTTGCCGTTCACGAAGCCAGCCAGGTTTCAGAGCTAGAAATAGCAAGTTG-3'	KH285: 5'-ATACCGCTCGAAGACCTGAGGAGACATAAAAAACAAAAAAAGCACCGACTCGGTGCC-3'
AG pSNR52	Gibson	PY122:BsaI cut; Chloramphenicol; -Leu; 2μ	pSNR52-pHTA1-C	KH465: 5'-AGGTGAGCCAGTGAGTTGATTGGAAGACCTGTGCGTGCTGGGTTGCGGC-3'	KH466: 5'- GCTCTGAAACCTGGCTGGCTTCGTGAACGGCAAGCACTGAAAATTTTTCGCCGCC-3'
			pHTA-C-gRNA-tSUP4	KH462: 5'-CTTGCCGTTCACGAAGCCAGCCAGGTTTCAGAGCTAGAAATAGCAAGTTG-3'	KH285: 5'-ATACCGCTCGAAGACCTGAGGAGACATAAAAAACAAAAAAAGCACCGACTCGGTGCC-3'
AG pSCR1	Gibson	PY122:BsaI cut; Chloramphenicol; -Leu; 2μ	pSCR1-pHTA1-C	KH463: 5'-GAGCCAGTGAGTTGATTGGAAGACCTGTGCCCTACTGTGCGTCACGTCTG-3'	KH463: 5'-AGCTCTGAAACCTGGCTGGCTTCGTGAACGGCAAGACCAGTGTGGTATCATGGCG-3'
			pHTA1-C-gRNA-tSUP4	KH462: 5'-CTTGCCGTTCACGAAGCCAGCCAGGTTTCAGAGCTAGAAATAGCAAGTTG-3'	KH285: 5'-ATACCGCTCGAAGACCTGAGGAGACATAAAAAACAAAAAAAGCACCGACTCGGTGCC-3'
CA pRPR1	Gibson	PY122:BsaI cut; Chloramphenicol; -Leu; 2μ	pRPR1-pHTA1-C	KH467: 5'- GGTGAGCCAGTGAGTTGATTGGAAGACCTGTGCCCCTGTTCAGGCGGTCAATTAG	KH468: 5'-CTCTGAAACCTGGCTGGCTTCGTGAACGGCAAGGTGCGATTACGCCAAAAGCTTG-3'
			pHTA1-C-gRNA-SUP4	KH462: 5'-CTTGCCGTTCACGAAGCCAGCCAGGTTTCAGAGCTAGAAATAGCAAGTTG-3'	KH285: 5'-ATACCGCTCGAAGACCTGAGGAGACATAAAAAACAAAAAAAGCACCGACTCGGTGCC-3'
CA pSCR1	Gibson	PY122:BsaI cut; Chloramphenicol; -Leu; 2μ	pSCR1-pHTA1-C	KH469: 5'- CAGTGAGTTGATTGGAAGACCTGTGCCTTTGGAGAAAGTTATTTTTATTCAAACCTAG-3'	KH470: 5'- CCTGGCTGGCTTCGTGAACGGCAAGCTTGAGATATATATTTTCTCAATAATGTTCATCTG-3'
			pHTA1-C-gRNA-tSUP4	KH462: CTTGCCGTTCACGAAGCCAGCCAGGTTTCAGAGCTAGAAATAGCAAGTTG-3'	KH285: 5'-ATACCGCTCGAAGACCTGAGGAGACATAAAAAACAAAAAAAGCACCGACTCGGTGCC-3'
CA pSNR52	Gibson	PY122:BsaI cut; Chloramphenicol; -Leu; 2μ	pSNR52	KH471: 5'-CCAGTGAGTTGATTGGAAGACCTGTGCATTACGTACCAAGAAGCATCTAATCAAC-3'	KH541: 5'-AGCGGTCTCACAAGAAATTAAAAATAGTTTACGCAAGTCTCGAAC-3'
			KanR Dummy-gRNA scaffold-tSUP4	KH540: 5'-CGAGACTTGCGTAAACTATTTTTAATTTCTTGTGAGACCGCTCGC-3'	KH285: 5'-ATACCGCTCGAAGACCTGAGGAGACATAAAAAACAAAAAAAGCACCGACTCGGTGCC-3'
KL pRPR1	Gibson	PY122:BsaI cut; Chloramphenicol; -Leu; 2μ	pRPR1	KH473: 5'- GAGTTGATTGGAAGACCTGTGCGGAGTTGATAATTTACATAAGTTTAATTATATTCTCG-3'	KH617: 5'- GCGAGCGGTCTCACAAGATTCGAACCGAAGTCGCAAC-3'
			KanR Dummy-gRNA scaffold-tSUP4	KH618: 5'- GTTGCGACTTCGGTTCGAATCTTGTGAGACCGCTCGC-3'	KH285: 5'-ATACCGCTCGAAGACCTGAGGAGACATAAAAAACAAAAAAAGCACCGACTCGGTGCC-3'
KL pSCR1	Gibson	PY122:BsaI cut; Chloramphenicol; -Leu; 2μ	pSCR1-pHTA1-C	KH475: 5'- GTGAGCCAGTGAGTTGATTGGAAGACCTGTGCGGCATGGCTGCATGAAGTATATC-3'	KH476: 5'- CTCTGAAACCTGGCTGGCTTCGTGAACGGCAAGTTCGACAGGGTGCTTTCTTAAAC
			pHTA1-C-gRNA-tSUP4	KH462: 5'- CTTGCCGTTCACGAAGCCAGCCAGGTTTCAGAGCTAGAAATAGCAAGTTG-3'	KH285: 5'-ATACCGCTCGAAGACCTGAGGAGACATAAAAAACAAAAAAAGCACCGACTCGGTGCC-3'
KL pSNR52	Gibson	PY122:BsaI cut; Chloramphenicol; -Leu; 2μ	pSNR52	KH619: 5'- TGAGCCAGTGAGTTGATTGGAAGACCTGTGCAATGAGGGAAAGAGGGAAATCG-3'	KH493: 5'- GCGAGCGGTCTCACAAGGATTCGAACTGCCGAGAAAGTAAC-3'
			KanR Dummy-gRNA scaffold-tSUP4	KH494: 5'-GTTACTTTCTCGGCAGTTCGAATCCTTGTGAGACCGCTCGC-3'	KH285: 5'-ATACCGCTCGAAGACCTGAGGAGACATAAAAAACAAAAAAAGCACCGACTCGGTGCC-3'
SC pSCR1	Gibson	PY122:BsaI cut; Chloramphenicol; -Leu; 2μ	pSCR1-pHTA1-C	KH310: 5'-GTTGATTGGAAGACCTGTGCCCAGGAACTTTTCATTGAAAATCTTCTG-3'	KH412: 5'-AACCTGGCTGGCTTCGTGAACGGAGCACAATTGGAATAAACTTTCGATTTTATAC-3'
			pHTA1-C-gRNA-tSUP4	KH413: 5'-AATTGTGCTCCGTTCACGAAGCCAGCCAGGTTTCAGAGCTAGAAATAGCAAGTTG-3'	KH285: 5'-ATACCGCTCGAAGACCTGAGGAGACATAAAAAACAAAAAAAGCACCGACTCGGTGCC-3'
SC Extended pSCR1	Gibson	PY122:BsaI cut; Chloramphenicol; -Leu; 2μ	pSCR1-pHTA1-C	KH320: 5'-GTTGATTGGAAGACCTGTGCTCCTTGTTATAACAACCCTCGG-3'	KH416: 5'-AACCTGGCTGGCTTCGTGAACGGAGCACAATTGGAATAAACTTTCGATTTTATAC-3'
			pHTA1-C-gRNA-tSUP4	KH417: 5'-AATTGTGCTCCGTTCACGAAGCCAGCCAGGTTTCAGAGCTAGAAATAGCAAGTTG-3'	KH285: 5'-ATACCGCTCGAAGACCTGAGGAGACATAAAAAACAAAAAAAGCACCGACTCGGTGCC-3'
SC pU6	Gibson	PY122:BsaI cut; Chloramphenicol; -Leu; 2μ	pU6-pHTA1-C	KH404: 5'-GTTGATTGGAAGACCTGTGCGTTTCAACACAGCCTGGC-3'	KH405: 5'-AAACCTGGCTGGCTTCGTGAACGGACATAGTTGCGAAAAAAACATTTATTTATAG-3'
			pHTA1-C-gRNA-tSUP4	KH406: 5'-CAACTATGTCCGTTCACGAAGCCAGCCAGGTTTCAGAGCTAGAAATAGCAAGTTG-3'	KH285: 5'-ATACCGCTCGAAGACCTGAGGAGACATAAAAAACAAAAAAAGCACCGACTCGGTGCC-3'
SC pRNA170	Gibson	PY122:BsaI cut; Chloramphenicol; -Leu; 2μ	pRNA170-pHTA1-C	KH407: 5'-GTTGATTGGAAGACCTGTGCGGCTATAATGGTCTGGGCTAGTC-3'	KH408: 5'-CTGAAACCTGGCTGGCTTCGTGAACGGTGGTATATAAGAGAAAGAAGCGGTAACG-3'
			pHTA1-C-gRNA-tSUP4	KH409: 5'-TATATACCACCGTTCACGAAGCCAGCCAGGTTTCAGAGCTAGAAATAGCAAGTTG-3'	KH285: 5'-ATACCGCTCGAAGACCTGAGGAGACATAAAAAACAAAAAAAGCACCGACTCGGTGCC-3'
Human pU6	Gibson	PY122:BsaI cut; Chloramphenicol; -Leu; 2μ	pU6-pHTA1-C	KH301: 5'-GTTGATTGGAAGACCTGTGCGAGGGCCTATTTCCCATGATTC-3'	KH426: 5'-CTCTGAAACCTGGCTGGCTTCGTGAACGGGTCCTTTCCACAAGATATATAAAGCC-3'
			pHTA1-C-gRNA-tSUP4	KH427: 5'-GGAAAGGACCCGTTCACGAAGCCAGCCAGGTTTCAGAGCTAGAAATAGCAAGTTG-3'	KH285: 5'-ATACCGCTCGAAGACCTGAGGAGACATAAAAAACAAAAAAAGCACCGACTCGGTGCC-3'
Mouse pU6	Gibson	PY122:BsaI cut; Chloramphenicol; -Leu; 2μ	pU6-pHTA1-C	KH296: 5'-GTTGATTGGAAGACCTGTGCGATCCGACGCCGCC-3'	KH424: 5'-TCTAGCTCTGAAACCTGGCTGGCTTCGTGAACGGAACAAGGCTTTTCTCCAAGGG-3'
			pHTA1-C-gRNA-tSUP4	KH425: 5'-AGCCTTGTTCCGTTCACGAAGCCAGCCAGGTTTCAGAGCTAGAAATAGCAAGTTG-3'	KH285: 5'-ATACCGCTCGAAGACCTGAGGAGACATAAAAAACAAAAAAAGCACCGACTCGGTGCC-3'
SC pSNR52-HDV	Gibson	PY122:BsaI cut; Chloramphenicol; -Leu; 2μ	pSNR52-HDV-KanR Dummy-gRNA-tSUP4	KH272: 5'-GTTGATTGGAAGACCTGTGCTCTTTGAAAAGATAATGTATGATTATGCTTTC-3'	KH285: 5'-ATACCGCTCGAAGACCTGAGGAGACATAAAAAACAAAAAAAGCACCGACTCGGTGCC-3'
tRNA^{Pro}	Gibson	PY122:BsaI cut; Chloramphenicol; -Leu; 2μ	tRNA^{Pro} - KanR Dummy-gRNA-tSUP4	KH1054: 5'-GTGAGTTGATTGGAAGACCTGTGCTCCGTAGTCTAAATGAGTTACGTACG-3'	KH285: 5'-ATACCGCTCGAAGACCTGAGGAGACATAAAAAACAAAAAAAGCACCGACTCGGTGCC-3'
tRNA^{Pro} -HDV	Gibson	PY122:BsaI cut; Chloramphenicol; -Leu; 2μ	tRNA^{Pro} - HDV-KanR Dummy-gRNA-tSUP4	KH1054: 5'-GTGAGTTGATTGGAAGACCTGTGCTCCGTAGTCTAAATGAGTTACGTACG-3'	KH285: 5'-ATACCGCTCGAAGACCTGAGGAGACATAAAAAACAAAAAAAGCACCGACTCGGTGCC-3'
tRNA^{Tyr}	Gibson	PY122:BsaI cut; Chloramphenicol; -Leu; 2μ	tRNA^{Tyr} -KanR Dummy-gRNA-tSUP4	KH1055: 5'-GTGAGTTGATTGGAAGACCTGTGCGTATACTCTTTCTTCAACAATTAAATACTCTCGG-3'	KH285: 5'-ATACCGCTCGAAGACCTGAGGAGACATAAAAAACAAAAAAAGCACCGACTCGGTGCC-3'
tRNA^{Tyr} -HDV	Gibson	PY122:BsaI cut; Chloramphenicol; -Leu; 2μ	tRNA^{Tyr} -HDV-KanR Dummy-gRNA-tSUP4	KH1055: 5'-GTGAGTTGATTGGAAGACCTGTGCGTATACTCTTTCTTCAACAATTAAATACTCTCGG-3'	KH285: 5'-ATACCGCTCGAAGACCTGAGGAGACATAAAAAACAAAAAAAGCACCGACTCGGTGCC-3'
KL pRPR1-HDV	Gibson	PY122:BsaI cut; Chloramphenicol; -Leu; 2μ	pRPR1-HDV-KanR Dummy-gRNA-tSUP4	KH473: 5'-GAGTTGATTGGAAGACCTGTGCGGAGTTGATAATTTACATAAGTTTAATTATATTCTCG-3'	KH285: 5'-ATACCGCTCGAAGACCTGAGGAGACATAAAAAACAAAAAAAGCACCGACTCGGTGCC-3'
KL pSNR52-HDV	Gibson	PY122:BsaI cut; Chloramphenicol; -Leu; 2μ	pSNR52-HDV-KanR Dummy-gRNA-tSUP4	KH619: 5'-TGAGCCAGTGAGTTGATTGGAAGACCTGTGCAATGAGGGAAAGAGGGAAATCG-3'	KH285: 5'-ATACCGCTCGAAGACCTGAGGAGACATAAAAAACAAAAAAAGCACCGACTCGGTGCC-3'

Supplemental Table S5: Assembly Method and Primer Sequences for 1X Pol III Promoter cassettes and Aptamer-scaffolded sgRNAs

1X Pol III Promoter Cassette Plasmids (MS2 Aptamers)	Assembly Method	Backbone	Fragments	Forward Primer	Reverse Primer
SC pSNR52	Gibson	PY122:BsaI cut; Chloramphenicol; -Leu; 2µ	pSNR52-KanR Dummy-MS21,3-tSUP4	KH272: 5'-GTTGATTGGAAGACCTGTGCTCTTTGAAAAGATAATGTATGATTATGCTTTC-3'	KH285: 5'-ATACCGCTCGAAGACCTGAGGAGACATAAAAAACAAAAAAAGCACCGACTCGGTGCC-3'
SC pRPR1	Gibson	PY122:BsaI cut; Chloramphenicol; -Leu; 2µ	pRPR1	KH315: 5'- GTTGATTGGAAGACCTGTGCGATCTGCCAATTGAACATAACATGG-3'	KH497: 5'-GCGAGCGGTCTCACAAGTGCCAATCGCAGCTCC-3'
			KanR Dummy-gRNA MS2 1,3-tSUP4	KH498: 5'- GGAGCTGCGATTGGCACTTGTGAGACCGCTCGC-3'	KH285: 5'-ATACCGCTCGAAGACCTGAGGAGACATAAAAAACAAAAAAAGCACCGACTCGGTGCC-3'
SC pZOD1	Gibson	PY122:BsaI cut; Chloramphenicol; -Leu; 2µ	pZOD1	KH428: 5'-GTTGATTGGAAGACCTGTGCCGTTGCATTACGTGGTGTCC-3'	KH489: 5'-GCGAGCGGTCTCACAAGATTTCCCGGCTTTTAAAATGCG-3'
			KanR Dummy-gRNA MS2 1,3-tSUP4	KH490: 5'-CGCATTTTAAAAGCCGGGAAATCTTGTGAGACCGCTCGC-3'	KH285: 5'-ATACCGCTCGAAGACCTGAGGAGACATAAAAAACAAAAAAAGCACCGACTCGGTGCC-3'
YL pSNR52	Gibson	PY122:BsaI cut; Chloramphenicol; -Leu; 2µ	pSNR52	KH334: 5'-GTTGATTGGAAGACCTGTGCGACCCCGTCTTCAATTACACTTC-3'	KH491: 5'-GCGAGCGGTCTCACAAGACGTCATTATTTTGCGTTAAGTTTCTAATC-3'
			KanR Dummy-gRNA MS2 1,3-tSUP4	KH492: 5'-GATTAGAAACTTAACGCAAAATAATGACGTCTTGTGAGACCGCTCGC-3'	KH285: 5'-ATACCGCTCGAAGACCTGAGGAGACATAAAAAACAAAAAAAGCACCGACTCGGTGCC-3'
YL pRPR1	Gibson	PY122:BsaI cut; Chloramphenicol; -Leu; 2µ	pRPR1	KH479: 5'-GTGAGTTGATTGGAAGACCTGTGCTATATATTTTTTATTCTAATGATTCATTACAGCTATATACC-3'	KH495: 5'-GCGAGCGGTCTCACAAGGGGTAACTGCAGTCTGACC-3'
			KanR Dummy-gRNA MS2 1,3-tSUP4	KH496: 5'- GGTCAGACTGCAGTTACCCCTTGTGAGACCGCTCGC-3'	KH285: 5'-ATACCGCTCGAAGACCTGAGGAGACATAAAAAACAAAAAAAGCACCGACTCGGTGCC-3'
CA pSNR52	Gibson	PY122:BsaI cut; Chloramphenicol; -Leu; 2µ	pSNR52	KH471: 5'-CCAGTGAGTTGATTGGAAGACCTGTGCATTACGTACCAAGAAGCATCTAATCAAC-3'	KH541: 5'-AGCGGTCTCACAAGAAATTAAAAATAGTTTACGCAAGTCTCGAAC-3'
			KanR Dummy-gRNA MS2 1,3-tSUP4	KH540: 5'-CGAGACTTGCGTAAACTATTTTTAATTTCTTGTGAGACCGCTCGC-3'	KH285: 5'-ATACCGCTCGAAGACCTGAGGAGACATAAAAAACAAAAAAAGCACCGACTCGGTGCC-3'
KL pRPR1	Gibson	PY122:BsaI cut; Chloramphenicol; -Leu; 2µ	pRPR1	KH473: 5'-GAGTTGATTGGAAGACCTGTGCGGAGTTGATAATTTACATAAGTTTAATTATATTCTCG-3'	KH617: 5'-GCGAGCGGTCTCACAAGATTCGAACCGAAGTCGCAAC-3'
			KanR Dummy-gRNA MS2 1,3-tSUP4	KH618: 5'-GTTGCGACTTCGGTTCGAATCTTGTGAGACCGCTCGC-3'	KH285: 5'-ATACCGCTCGAAGACCTGAGGAGACATAAAAAACAAAAAAAGCACCGACTCGGTGCC-3'
KL pSNR52	Gibson	PY122:BsaI cut; Chloramphenicol; -Leu; 2µ	pSNR52	KH619: 5'-TGAGCCAGTGAGTTGATTGGAAGACCTGTGCAATGAGGGAAAGAGGGAAATCG-3'	KH493: 5'-GCGAGCGGTCTCACAAGGATTCGAACTGCCGAGAAAGTAAC
			KanR Dummy-gRNA MS2 1,3-tSUP4	KH494: 5'-GTTACTTTCTCGGCAGTTCGAATCCTTGTGAGACCGCTCGC-3'	KH285: 5'-ATACCGCTCGAAGACCTGAGGAGACATAAAAAACAAAAAAAGCACCGACTCGGTGCC-3'
KL pSNR52-HDV	Gibson	PY122:BsaI cut; Chloramphenicol; -Leu; 2µ	KL pSNR52-HDV-pHTA1 C	KH619: 5'-TGAGCCAGTGAGTTGATTGGAAGACCTGTGCAATGAGGGAAAGAGGGAAATCG-3'	KH1136: 5'-TCTGAAACCTGGCTGGCTTCGTGAACGGCAAGAAAGTCCCATTCGCCACC-3'
			pHTA1 C-gRNA MS2 1,3-tSUP4	KH1137: 5'-CTTGCCGTTCACGAAGCCAGCCAGGTTTCAGAGCTAGGCCAACATG-3'	KH285: 5'-ATACCGCTCGAAGACCTGAGGAGACATAAAAAACAAAAAAAGCACCGACTCGGTGCC-3'
tRNA^{Pro}	Gibson	PY122:BsaI cut; Chloramphenicol; -Leu; 2µ	tRNA^{Pro} -pHTA1 C	KH1054: 5'-GTGAGTTGATTGGAAGACCTGTGCTCCGTAGTCTAAATGAGTTACGTACG-3'	KH1140: 5'-TCTGAAACCTGGCTGGCTTCGTGAACGGCAAGGGGGCGAGCTGGGAATTG-3'
			pHTA1 C-gRNA MS2 1,3-tSUP4	KH1137: 5'-CTTGCCGTTCACGAAGCCAGCCAGGTTTCAGAGCTAGGCCAACATG-3'	KH285: 5'-ATACCGCTCGAAGACCTGAGGAGACATAAAAAACAAAAAAAGCACCGACTCGGTGCC-3'
tRNA^{Pro}-HDV	Gibson	PY122:BsaI cut; Chloramphenicol; -Leu; 2µ	tRNA^{Pro}-HDV-pHTA1 C	KH1054: 5'-GTGAGTTGATTGGAAGACCTGTGCTCCGTAGTCTAAATGAGTTACGTACG-3'	KH1136: 5'-TCTGAAACCTGGCTGGCTTCGTGAACGGCAAGAAAGTCCCATTCGCCACC-3'
			pHTA1 C-gRNA MS2 1,3-tSUP4	KH1137: 5'-CTTGCCGTTCACGAAGCCAGCCAGGTTTCAGAGCTAGGCCAACATG-3'	KH285: 5'-ATACCGCTCGAAGACCTGAGGAGACATAAAAAACAAAAAAAGCACCGACTCGGTGCC-3'
**1X Pol III Pomoter Cassette Plasmids (PP7 Aptamers)**	**Assembly Method**	**Backbone**	**Fragments**	**Forward Primer**	**Reverse Primer**
SC pSNR52	Gibson	PY122:BsaI cut; Chloramphenicol; -Leu; 2µ	pSNR52-KanR Dummy	KH272: 5'-GTTGATTGGAAGACCTGTGCTCTTTGAAAAGATAATGTATGATTATGCTTTC-3'	KH213: 5'-GGTAGCTCTGAAACTGAGACCGAAGGAGAAAACTCACCGAGG-3'
			PP71,3_tSUP4	KH214: 5'-GGTGAGTTTTCTCCTTCGGTCTCAGTTTCAGAGCTACCGGAGCAGAC-3'	KH285: 5'-ATACCGCTCGAAGACCTGAGGAGACATAAAAAACAAAAAAAGCACCGACTCGGTGCC-3'
SC pRPR1	Gibson	PY122:BsaI cut; Chloramphenicol; -Leu; 2µ	pRPR1	KH315: 5'- GTTGATTGGAAGACCTGTGCGATCTGCCAATTGAACATAACATGG-3'	KH497: 5'-GCGAGCGGTCTCACAAGTGCCAATCGCAGCTCC-3'
			KanR Dummy-gRNA PP71,3-tSUP4	KH498: 5'- GGAGCTGCGATTGGCACTTGTGAGACCGCTCGC-3'	KH285: 5'-ATACCGCTCGAAGACCTGAGGAGACATAAAAAACAAAAAAAGCACCGACTCGGTGCC-3'
SC pZOD1	Gibson	PY122:BsaI cut; Chloramphenicol; -Leu; 2µ	pZOD1	KH428: 5'-GTTGATTGGAAGACCTGTGCCGTTGCATTACGTGGTGTCC-3'	KH489: 5'-GCGAGCGGTCTCACAAGATTTCCCGGCTTTTAAAATGCG-3'
			KanR Dummy-gRNA PP71,3-tSUP4	KH490: 5'-CGCATTTTAAAAGCCGGGAAATCTTGTGAGACCGCTCGC-3'	KH285: 5'-ATACCGCTCGAAGACCTGAGGAGACATAAAAAACAAAAAAAGCACCGACTCGGTGCC-3'
YL pSNR52	Gibson	PY122:BsaI cut; Chloramphenicol; -Leu; 2µ	pSNR52	KH334: 5'-GTTGATTGGAAGACCTGTGCGACCCCGTCTTCAATTACACTTC-3'	KH491: 5'-GCGAGCGGTCTCACAAGACGTCATTATTTTGCGTTAAGTTTCTAATC-3'
			KanR Dummy-gRNA PP71,3-tSUP4	KH492: 5'-GATTAGAAACTTAACGCAAAATAATGACGTCTTGTGAGACCGCTCGC-3'	KH285: 5'-ATACCGCTCGAAGACCTGAGGAGACATAAAAAACAAAAAAAGCACCGACTCGGTGCC-3'
YL pRPR1	Gibson	PY122:BsaI cut; Chloramphenicol; -Leu; 2µ	pRPR1	KH479: 5'-GTGAGTTGATTGGAAGACCTGTGCTATATATTTTTTATTCTAATGATTCATTACAGCTATATACC-3'	KH495: 5'-GCGAGCGGTCTCACAAGGGGTAACTGCAGTCTGACC-3'
			KanR Dummy-gRNA PP71,3-tSUP4	KH496: 5'- GGTCAGACTGCAGTTACCCCTTGTGAGACCGCTCGC-3'	KH285: 5'-ATACCGCTCGAAGACCTGAGGAGACATAAAAAACAAAAAAAGCACCGACTCGGTGCC-3'
CA pSNR52	Gibson	PY122:BsaI cut; Chloramphenicol; -Leu; 2µ	pSNR52	KH471: 5'-CCAGTGAGTTGATTGGAAGACCTGTGCATTACGTACCAAGAAGCATCTAATCAAC-3'	KH541: 5'-AGCGGTCTCACAAGAAATTAAAAATAGTTTACGCAAGTCTCGAAC-3'
			KanR Dummy-gRNA PP71,3-tSUP4	KH540: 5'-CGAGACTTGCGTAAACTATTTTTAATTTCTTGTGAGACCGCTCGC-3'	KH285: 5'-ATACCGCTCGAAGACCTGAGGAGACATAAAAAACAAAAAAAGCACCGACTCGGTGCC-3'
KL pRPR1	Gibson	PY122:BsaI cut; Chloramphenicol; -Leu; 2µ	pRPR1	KH473: 5'-GAGTTGATTGGAAGACCTGTGCGGAGTTGATAATTTACATAAGTTTAATTATATTCTCG-3'	KH617: 5'-GCGAGCGGTCTCACAAGATTCGAACCGAAGTCGCAAC-3'
			KanR Dummy-gRNA PP7,3-tSUP4	KH618: 5'-GTTGCGACTTCGGTTCGAATCTTGTGAGACCGCTCGC-3'	KH285: 5'-ATACCGCTCGAAGACCTGAGGAGACATAAAAAACAAAAAAAGCACCGACTCGGTGCC-3'
KL pSNR52	Gibson	PY122:BsaI cut; Chloramphenicol; -Leu; 2µ	pSNR52	KH619: 5'-TGAGCCAGTGAGTTGATTGGAAGACCTGTGCAATGAGGGAAAGAGGGAAATCG-3'	KH493: 5'-GCGAGCGGTCTCACAAGGATTCGAACTGCCGAGAAAGTAAC
			KanR Dummy-gRNA PP71,3-tSUP4	KH494: 5'-GTTACTTTCTCGGCAGTTCGAATCCTTGTGAGACCGCTCGC-3'	KH285: 5'-ATACCGCTCGAAGACCTGAGGAGACATAAAAAACAAAAAAAGCACCGACTCGGTGCC-3'
KL pRPR1-HDV	Gibson	PY122:BsaI cut; Chloramphenicol; -Leu; 2µ	KL pRPR1-HDV-pAgTEF1 F	KH473: 5'-GAGTTGATTGGAAGACCTGTGCGGAGTTGATAATTTACATAAGTTTAATTATATTCTCG-3'	KH1138: 5'-TGAAACTGCTCGCAGGTCTGCAGCGACAAGAAAGTCCCATTCGCCACC-3'
			pAgTEF1 F-gRNA PP7 1,3-tSUP4	KH1139: 5'-CTTGTCGCTGCAGACCTGCGAGCAGTTTCAGAGCTACCGGAGC-3'	KH285: 5'-ATACCGCTCGAAGACCTGAGGAGACATAAAAAACAAAAAAAGCACCGACTCGGTGCC-3'
tRNA^{Tyr}	Gibson	PY122:BsaI cut; Chloramphenicol; -Leu; 2µ	tRNA^{Tyr} -pAgTEF1 F	KH1055: 5'-GTGAGTTGATTGGAAGACCTGTGCGTATACTCTTTCTTCAACAATTAAATACTCTCGG-3'	KH1143: 5'-GCTCTGAAACTGCTCGCAGGTCTGCAGCGACAAGTCTCCCGGGGGCG-3'
			pAgTEF1 F-gRNA PP7 1,3-tSUP4	KH1139: 5'-CTTGTCGCTGCAGACCTGCGAGCAGTTTCAGAGCTACCGGAGC-3'	KH285: 5'-ATACCGCTCGAAGACCTGAGGAGACATAAAAAACAAAAAAAGCACCGACTCGGTGCC-3'
tRNA^{Tyr}-HDV	Gibson	PY122:BsaI cut; Chloramphenicol; -Leu; 2µ	tRNA^{Tyr}-HDV-pAgTEF1 F	KH1055: 5'-GTGAGTTGATTGGAAGACCTGTGCGTATACTCTTTCTTCAACAATTAAATACTCTCGG-3'	KH1144: 5'-CTGAAACTGCTCGCAGGTCTGCAGCGACAAGAAAGTCCCATTCGCCACC-3'
			pAgTEF1 F-gRNA PP7 1,3-tSUP4	KH1139: 5'-CTTGTCGCTGCAGACCTGCGAGCAGTTTCAGAGCTACCGGAGC-3'	KH285: 5'-ATACCGCTCGAAGACCTGAGGAGACATAAAAAACAAAAAAAGCACCGACTCGGTGCC-3'

Supplemental Table S6: Assembly Method and Primer Sequences for 2X and 3X Pol III Promoter cassettes and Aptamer-scaffolded sgRNAs

2X Pol III Promoter Cassette Plasmids	Assembly Method	Backbone	Fragments	Forward Primer	Reverse Primer
(KL pSNR52-pHTA1-C-MS2 1,3-tSUP4)-(KL pRPR1-AgTEF1-F-PP71,3-tSUP4)_AX-A	Gibson	PY122:BsaI cut; Chloramphenicol; -Leu; 2µ	KL pSNR52-pHTA1-C-MS21,3-tSUP4	KH619: 5'-TGAGCCAGTGAGTTGATTGGAAGACCTGTGCAATGAGGGAAAGAGGGAAATCG-3'	KH693: 5'-AATATAATTAAACTTATGTAAATTATCAACTCCAGACATAAAAAACAAAAAAAGCACCG-3'
			KL pRPR1-pAgTEF1-F-PP71,3-tSUP4	KH694: 5'-GTGCTTTTTTTGTTTTTTATGTCTGGAGTTGATAATTTACATAAGTTTAATTATATTCTCGTCC-3'	KH285: 5'-ATACCGCTCGAAGACCTGAGGAGACATAAAAAACAAAAAAAGCACCGACTCGGTGCC-3'
(KL pRPR1-pHTA1-C-MS2 1,3-tSUP4)-(KL pSNR52-AgTEF1 F-PP7 1,3-tSUP4)_AX-B	Gibson	PY122:BsaI cut; Chloramphenicol; -Leu; 2µ	KL pRPR1-pHTA1-C-MS21,3-tSUP4	KH473: 5'-GAGTTGATTGGAAGACCTGTGCGGAGTTGATAATTTACATAAGTTTAATTATATTCTCG-3'	KH695: 5'-AACGATTTCCCTCTTTCCCTCATTAGACATAAAAAACAAAAAAAGCACCG-3'
			KL pSNR52-pAgTEF1-F-PP71,3-tSUP4	KH696: 5'-CGGTGCTTTTTTTGTTTTTTATGTCTAATGAGGGAAAGAGGGAAATCGTTATG-3'	KH285: 5'-ATACCGCTCGAAGACCTGAGGAGACATAAAAAACAAAAAAAGCACCGACTCGGTGCC-3'
(SC pSNR52-pHTA1-C-MS2 1,3-tSUP4)-(SC pRPR1-AgTEF1-F-PP7 1,3-tSUP4)_BS_H	Gibson	PY122:BsaI cut; Chloramphenicol; -Leu; 2µ	SC pSNR52-pHTA1-C-MS21,3-tSUP4	KH732: 5'-TGAGCCAGTGAGTTGATTGGAAGACCTGTGCTCTTTGAAAAGATAATGTATGATTATGCTTTCAC-3'	KH733: 5'-CATGTTATGTTCAATTGGCAGATCAGACATAAAAAACAAAAAAAGCACCG-3'
			SC pRPR1-pAgTEF1-F-PP71,3-tSUP4	KH734: 5'-GCTTTTTTTGTTTTTTATGTCTGATCTGCCAATTGAACATAACATGGTAG-3'	KH285: 5'-ATACCGCTCGAAGACCTGAGGAGACATAAAAAACAAAAAAAGCACCGACTCGGTGCC-3'
(SC pSNR52-pHTA1-C-PP7 1,3-tSUP4)-(SC pRPR1-AgTEF1-F-MS2 1,3-tSUP4)_BS_G	Gibson	PY122:BsaI cut; Chloramphenicol; -Leu; 2µ	SC pSNR52-pHTA1-C-PP71,3-tSUP4	KH732: 5'-TGAGCCAGTGAGTTGATTGGAAGACCTGTGCTCTTTGAAAAGATAATGTATGATTATGCTTTCAC-3'	KH733: 5'-CATGTTATGTTCAATTGGCAGATCAGACATAAAAAACAAAAAAAGCACCG-3'
			SC pRPR1-pAgTEF1-F-MS21,3-tSUP4	KH734: 5'-GCTTTTTTTGTTTTTTATGTCTGATCTGCCAATTGAACATAACATGGTAG-3'	KH285: 5'-ATACCGCTCGAAGACCTGAGGAGACATAAAAAACAAAAAAAGCACCGACTCGGTGCC-3'
(SC pSNR52-CTT1-MS2 1,3-tSUP4)-(SC pRPR1-TKL1-PP7 1,3-tSUP4)_KA_A	Gibson	PY122:BsaI cut; Chloramphenicol; -Leu; 2µ	SC pSNR52- CTT1-MS21,3-tSUP4	KH732: 5'-TGAGCCAGTGAGTTGATTGGAAGACCTGTGCTCTTTGAAAAGATAATGTATGATTATGCTTTCAC-3'	KH733: 5'-CATGTTATGTTCAATTGGCAGATCAGACATAAAAAACAAAAAAAGCACCG-3'
			SC pRPR1-TKL1-PP71,3-tSUP4	KH734: 5'-GCTTTTTTTGTTTTTTATGTCTGATCTGCCAATTGAACATAACATGGTAG-3'	KH285: 5'-ATACCGCTCGAAGACCTGAGGAGACATAAAAAACAAAAAAAGCACCGACTCGGTGCC-3'
(KL pSNR52-CTT1-MS2 1,3-tSUP4)-(KL pRPR1-TKL1-PP7 1,3-tSUP4)_KA_C	Gibson	PY122:BsaI cut; Chloramphenicol; -Leu; 2µ	KL pSNR52-CTT1-MS21,3-tSUP4	KH619: 5'-TGAGCCAGTGAGTTGATTGGAAGACCTGTGCAATGAGGGAAAGAGGGAAATCG-3'	KH693: 5'-AATATAATTAAACTTATGTAAATTATCAACTCCAGACATAAAAAACAAAAAAAGCACCG-3'
			KL pRPR1-TKL1-PP71,3-tSUP4	KH694: 5'-GTGCTTTTTTTGTTTTTTATGTCTGGAGTTGATAATTTACATAAGTTTAATTATATTCTCGTCC-3'	KH285: 5'-ATACCGCTCGAAGACCTGAGGAGACATAAAAAACAAAAAAAGCACCGACTCGGTGCC-3'
(tRNA^{Pro}-pHTA1 C-MS2 1,3-tSUP4)-(tRNA^{Tyr}-pAgTEF1 F-PP7 1,3-tSUP4)_RV_H	Gibson	PY122:BsaI cut; Chloramphenicol; -Leu; 2µ	tRNA^{Pro}-pHTA1 C	KH1054: 5'-GTGAGTTGATTGGAAGACCTGTGCTCCGTAGTCTAAATGAGTTACGTACG-3'	KH1140: 5'-TCTGAAACCTGGCTGGCTTCGTGAACGGCAAGGGGGCGAGCTGGGAATTG-3'
			pHTA1 C- MS2 1,3-tSUP4	KH1137: 5'-CTTGCCGTTCACGAAGCCAGCCAGGTTTCAGAGCTAGGCCAACATG-3'	KH1141: 5'-TTGTTGAAGAAAGAGTATACAGACATAAAAAACAAAAAAAGCACCGACTCGGTGC-3'
			tSUP4-tRNA^{Tyr}-pAgTEF1 F	KH1142: 5'-GTGCTTTTTTTGTTTTTTATGTCTGTATACTCTTTCTTCAACAATTAAATACTCTC-3'	KH1143: 5'-GCTCTGAAACTGCTCGCAGGTCTGCAGCGACAAGTCTCCCGGGGGCG-3'
			pAgTEF1 F-PP7 1,3-tSUP4	KH1139: 5'-CTTGTCGCTGCAGACCTGCGAGCAGTTTCAGAGCTACCGGAGC-3'	KH285: 5'-ATACCGCTCGAAGACCTGAGGAGACATAAAAAACAAAAAAAGCACCGACTCGGTGCC-3'
(tRNA^{Pro}-HDV-pHTA1 C-MS2 1,3-tSUP4)-(tRNA^{Tyr}-HDV-pAgTEF1 F-PP7 1,3-tSUP4) RV_I	Gibson	PY122:BsaI cut; Chloramphenicol; -Leu; 2µ	tRNA^{Pro}-HDV-pHTA1 C	KH1054: 5'-GTGAGTTGATTGGAAGACCTGTGCTCCGTAGTCTAAATGAGTTACGTACG-3'	KH1136: 5'-TCTGAAACCTGGCTGGCTTCGTGAACGGCAAGAAAGTCCCATTCGCCACC-3'
			pHTA1 C- MS2 1,3-tSUP4	KH1137: 5'-CTTGCCGTTCACGAAGCCAGCCAGGTTTCAGAGCTAGGCCAACATG-3'	KH1141: 5'-TTGTTGAAGAAAGAGTATACAGACATAAAAAACAAAAAAAGCACCGACTCGGTGC-3'
			tSUP4-tRNA^{Tyr}-HDV-pAgTEF1 F	KH1142: 5'-GTGCTTTTTTTGTTTTTTATGTCTGTATACTCTTTCTTCAACAATTAAATACTCTC-3'	KH1144: 5'-CTGAAACTGCTCGCAGGTCTGCAGCGACAAGAAAGTCCCATTCGCCACC-3'
			pAgTEF1 F-PP7 1,3-tSUP4	KH1139: 5'-CTTGTCGCTGCAGACCTGCGAGCAGTTTCAGAGCTACCGGAGC-3'	KH285: 5'-ATACCGCTCGAAGACCTGAGGAGACATAAAAAACAAAAAAAGCACCGACTCGGTGCC-3'
**3X Pol III Pomoter Cassette Plasmids**					
(SC pSNR52-pTDH3 Seq 1-gRNA-tSUP4)-(KL pSNR52-pTEF1 Seq 2-tSUP4)-(KL pRPR1-pPGK1 Seq 1-gRNA-tSUP4)_RV_J	Gibson	PY122:BsaI cut; Chloramphenicol; -Leu; 2µ	SC pSNR52-pTDH3 Seq 1-gRNA-tSUP4	KH732: 5'-TGAGCCAGTGAGTTGATTGGAAGACCTGTGCTCTTTGAAAAGATAATGTATGATTATGCTTTCAC-3'	KH1145: 5'-CGATTTCCCTCTTTCCCTCATTAGACATAAAAAACAAAAAAAGCACCGACTCGGTGCC-3'
			tSUP4-KL pSNR52-pTEF1 Seq 2-tSUP4	KH1146: 5'-GTCGGTGCTTTTTTTGTTTTTTATGTCTAATGAGGGAAAGAGGGAAATCG-3'	KH1147: 5'- TATGTAAATTATCAACTCCAGACATAAAAAACAAAAAAAGCACCGACTCGGTGCC-3'
			tSUP4-KL pRPR1-pPGK1 Seq1-gRNA-tSUP4	KH1148: 5'-TTTTTGTTTTTTATGTCTGGAGTTGATAATTTACATAAGTTTAATTATATTCTCG-3'	KH285: 5'-ATACCGCTCGAAGACCTGAGGAGACATAAAAAACAAAAAAAGCACCGACTCGGTGCC-3'
(SC pSNR52-HDV-pTDH3 Seq 1-gRNA-tSUP4)-(KL pSNR52-HDV-pTEF1 Seq 2-tSUP4)-(KL pRPR1-HDV-pPGK1 Seq 1-gRNA-tSUP4)_RV_K	Gibson	PY122:BsaI cut; Chloramphenicol; -Leu; 2µ	SC pSNR52-HDV-pTDH3 Seq 1-gRNA-tSUP4	KH732: 5'-TGAGCCAGTGAGTTGATTGGAAGACCTGTGCTCTTTGAAAAGATAATGTATGATTATGCTTTCAC-3'	KH1145: 5'-CGATTTCCCTCTTTCCCTCATTAGACATAAAAAACAAAAAAAGCACCGACTCGGTGCC-3'
			tSUP4-KL pSNR52-HDV-pTEF1 Seq 2-tSUP4	KH1146: 5'-GTCGGTGCTTTTTTTGTTTTTTATGTCTAATGAGGGAAAGAGGGAAATCG-3'	KH1147: 5'- TATGTAAATTATCAACTCCAGACATAAAAAACAAAAAAAGCACCGACTCGGTGCC-3'
			tSUP4-KL pRPR1-HDV-pPGK1 Seq1-gRNA-tSUP4	KH1148: 5'-TTTTTGTTTTTTATGTCTGGAGTTGATAATTTACATAAGTTTAATTATATTCTCG-3'	KH285: 5'-ATACCGCTCGAAGACCTGAGGAGACATAAAAAACAAAAAAAGCACCGACTCGGTGCC-3'
(SC pSNR52-pTDH3 Seq 1-gRNA-tSUP4)-(tRNA^{Pro}-pTEF1 Seq 2-tSUP4)-(tRNA^{Tyr}-pPGK1 Seq 1-gRNA-tSUP4)_RV_L	Gibson	PY122:BsaI cut; Chloramphenicol; -Leu; 2µ	SC pSNR52-pTDH3 Seq 1-gRNA-tSUP4	KH732: 5'-TGAGCCAGTGAGTTGATTGGAAGACCTGTGCTCTTTGAAAAGATAATGTATGATTATGCTTTCAC-3'	KH1149: 5'-GTAACTCATTTAGACTACGGAAGACATAAAAAACAAAAAAAGCACCGACTCGGTGCC-3'
			tSUP4-tRNA^{Pro}-pTEF1 Seq 2-tSUP4	KH1150: 5'-GTGCTTTTTTTGTTTTTTATGTCTTCCGTAGTCTAAATGAGTTACGTACG-3'	KH1151- 5'-TGTTGAAGAAAGAGTATACAGACATAAAAAACAAAAAAAGCACCGACTCGGTGCC-3'
			tSUP4-tRNA^{Tyr}-pPGK1 Seq1-gRNA-tSUP4	KH1152: 5'-CTTTTTTTGTTTTTTATGTCTGTATACTCTTTCTTCAACAATTAAATACTCTCG-3'	KH285: 5'-ATACCGCTCGAAGACCTGAGGAGACATAAAAAACAAAAAAAGCACCGACTCGGTGCC-3'
(SC pSNR52-HDV-pTDH3 Seq 1-gRNA-tSUP4)-(tRNA^{Pro}-HDV-pTEF1 Seq 2-tSUP4)-(tRNA^{Tyr}-HDV-pPGK1 Seq 1-gRNA-tSUP4)_RV_M	Gibson	PY122:BsaI cut; Chloramphenicol; -Leu; 2µ	SC pSNR52-pTDH3 Seq 1-gRNA-tSUP4	KH732: 5'-TGAGCCAGTGAGTTGATTGGAAGACCTGTGCTCTTTGAAAAGATAATGTATGATTATGCTTTCAC-3'	KH1149: 5'-GTAACTCATTTAGACTACGGAAGACATAAAAAACAAAAAAAGCACCGACTCGGTGCC-3'
			tSUP4-tRNA^{Pro}-HDV-pTEF1 Seq 2-tSUP4	KH1150: 5'-GTGCTTTTTTTGTTTTTTATGTCTTCCGTAGTCTAAATGAGTTACGTACG-3'	KH1151- 5'-TGTTGAAGAAAGAGTATACAGACATAAAAAACAAAAAAAGCACCGACTCGGTGCC-3'
			tSUP4-tRNA^{Tyr}-HDV-pPGK1 Seq1-gRNA-tSUP4	KH1152: 5'-CTTTTTTTGTTTTTTATGTCTGTATACTCTTTCTTCAACAATTAAATACTCTCG-3'	KH285: 5'-ATACCGCTCGAAGACCTGAGGAGACATAAAAAACAAAAAAAGCACCGACTCGGTGCC-3'

Supplemental Table S7: Assembly Method and Primer Sequences for 1X Pol III Promoter cassettes with Separate crRNA and tracrRNAs

Separate crRNA and tracrRNA Pol III Promoter Cassette Plasmids	Assembly Method	Backbone	Fragments	Forward Primer	Reverse Primer	CRISPR RNA (crRNA) Sequence	Trans-activating CRISPR RNA (tracrRNA) Sequences
SC pSNR52_KanR Dummy_crRNA_tSUP4 (BB_crRNA)	Gibson	PY122:BsaI cut; Chloramphenicol; -Leu; 2µ	pSNR52-KanR Dummy_crRNA_tSUP4	KH272: 5'-GTTGATTGGAAGACCTGTGCTCTTTGAAAAGATAATGTATGATTATGCTTTC-3'	KH327: [illegible]	GTTTTAGAGCTATGCTGTTTTG	N/A
KL pRPR1_gRNA_tSUP4 (AX_gRNA)	Gibson	PY128:BsaI cut; [illegible]; -Leu; 2µ	KL pRPR1	KH473: [illegible]	KH328: [illegible]	N/A	N/A
			gRNA_tSUP4	KH329: [illegible]	KH332: [illegible]	N/A	[illegible]
KL pRPR1_MS2 2X Tail_tSUP4 (AX_2XT)	Gibson	PY128:BsaI cut; [illegible]; -Leu; 2µ	KL pRPR1	KH473: [illegible]	KH328: [illegible]	N/A	N/A
			MS2 2X Tail_tSUP4	KH329: [illegible]	KH330: [illegible]	N/A	[illegible]
SC pSNR52_gRNA_tSUP4 (BS_gRNA)	Gibson	PY128:BsaI cut; [illegible]; -Leu; 2µ	SC SNR52	KH272: [illegible]	KH333: [illegible]	N/A	N/A
			gRNA_tSUP4	KH334: [illegible]	KH332: [illegible]	N/A	[illegible]
SC pSNR52_MS2 2X Tail_tSUP4 (BS_2XT)	Gibson	PY128:BsaI cut; [illegible]; -Leu; 2µ	SC SNR52	KH272: [illegible]	KH333: [illegible]	N/A	N/A
			MS2 2X Tail_tSUP4	KH334: [illegible]	KH330: [illegible]	N/A	[illegible]

Supplemental Table S8: 20 bps Targeting Sequences with BsaI Overhangs and RT-qPCR primers

Spacer Inserts	Oligo Sequences	Length
KH615 (pHTA1-A)	CTTGACTGTGCGAAGCTATTGGAA	24 bps
KH616 (pHTA1-A)	AAACTTCCAATAGCTTCGCACAGT	24 bps
KH207 (pHTA1-B)	CTTGTGAGAAACCACATATCTCTA	24 bps
KH208 (pHTA1-B)	AAACTAGAGATATGTGGTTTCTCA	24 bps
KH487 (pHTA1-C)	CTTGCCGTTCACGAAGCCAGCCAG	24 bps
KH488 (pHTA1-C)	AAACCTGGCTGGCTTCGTGAACGG	24 bps
KH209 (pHTA1-D)	CTTGATCTGTACTTCTCTTCTTGA	24 bps
KH210 (pHTA1-D)	AAACTCAAGAAGAGAAGTACAGAT	24 bps
KH439 (pAgtEF1-E)	CTTGCATACATTTTGATGGCCGCA	24 bps
KH440 (pAgtEF1-E)	AAACTGCGGCCATCAAAATGTATG	24 bps
KH441 (pAgtEF1-F)	CTTGTCGCTGCAGACCTGCGAGCA	24 bps
KH442 (pAgtEF1-F)	AAACTGCTCGCAGGTCTGCAGCGA	24 bps
KH443 (pAgtEF1-G)	CTTGGACAATTCAACGCGTCTGTG	24 bps
KH444 (pAgtEF1-G)	AAACCACAGACGCGTTGAATTGTC	24 bps
K074 (pTDH3 Seq 1)	CTTGCAAGTAGGGGAATAATTTCA	24 bps
KH075 (pTDH3 Seq 1)	AAACTGAAATTATTCCCCTACTTG	24 bps
KH082 (pTEF1 Seq 2)	CTTGTCGACGAAGAAAAAGAAACG	24 bps
KH083 (pTEF1 Seq 2)	AAACCGTTTCTTTTTCTTCGTCGA	24 bps
KH086 (pPGK1 Seq 1)	CTTGGCTGTTGTTGTCACACGATT	24 bps
KH087 (pPGK1 Seq 1)	AAACAATCGTGTGACAACAACAGC	24 bps
KH121 (NT1)	CTTGGTTTGGTTCTTTTTTCTTCC	24 bps
KH122 (NT1)	AAACGGAAGAAAAAAGAACCAAAC	24 bps
KH139 (NT2)	AAACCATGGACGTTAGGCTTGTTT	24 bps
KH140 (NT2)	CTTGCACCATTCGCGGTCAGGGGG	24 bps
KH141 (NT3)	AAACCCCCCTGACCGCGAATGGTG	24 bps
KH142 (NT3)	CTTGGAACCGTGGCGCGCTGGATG	24 bps
KH724 (CTT1)	CTTGGTATAGTTCACTTCACCCTC	24 bps
KH725 (CTT1)	AAACGAGGGTGAAGTGAACTATAC	24 bps
KH726 (TKL1)	CTTGATGTAGGTAAAAGATCAGGG	24 bps
KH727 (TKL1)	AAACCCCTGATCTTTTACCTACAT	24 bps
KH743 (ACT1)	CAATTCGTTGTAGAAGGTATGATGCC	26 bps
KH744 (ACT1)	ATTATATGTTTAGAGGTTGCTGCTTTGG	28 bps
